# Metabolomics Reveals the Heterogeneous Secretome of Two Entomopathogenic Fungi to *Ex Vivo* Cultured Insect Tissues

**DOI:** 10.1371/journal.pone.0070609

**Published:** 2013-08-05

**Authors:** Charissa de Bekker, Philip B. Smith, Andrew D. Patterson, David P. Hughes

**Affiliations:** 1 Department of Entomology and Department of Biology, Center for Infectious Disease Dynamics, Pennsylvania State University, University Park, State College, Pennsylvania, United States of America; 2 Metabolomics Core Facility, Huck Institutes of Life Sciences, Pennsylvania State University, University Park, State College, Pennsylvania, United States of America; 3 Center for Molecular Toxicology and Carcinogenesis, Department of Veterinary and Biomedical Sciences, Pennsylvania State University, University Park, State College, Pennsylvania, United States of America; Leibniz Institute for Natural Products Research and Infection Biology- Hans Knoell Institute, Germany

## Abstract

Fungal entomopathogens rely on cellular heterogeneity during the different stages of insect host infection. Their pathogenicity is exhibited through the secretion of secondary metabolites, which implies that the infection life history of this group of environmentally important fungi can be revealed using metabolomics. Here metabolomic analysis in combination with *ex vivo* insect tissue culturing shows that two generalist isolates of the genus *Metarhizium* and *Beauveria*, commonly used as biological pesticides, employ significantly different arrays of secondary metabolites during infectious and saprophytic growth. It also reveals that both fungi exhibit tissue specific strategies by a distinguishable metabolite secretion on the insect tissues tested in this study. In addition to showing the important heterogeneous nature of these two entomopathogens, this study also resulted in the discovery of several novel destruxins and beauverolides that have not been described before, most likely because previous surveys did not use insect tissues as a culturing system. While *Beauveria* secreted these cyclic depsipeptides when encountering live insect tissues, *Metarhizium* employed them primarily on dead tissue. This implies that, while these fungi employ comparable strategies when it comes to entomopathogenesis, there are most certainly significant differences at the molecular level that deserve to be studied.

## Introduction

Isogenic cell populations of both prokaryotic and eukaryotic organisms can exhibit a highly dynamic transcriptome, proteome and metabolome. In many cases cellular heterogeneity occurs in response to environmental stresses, functioning as an important mechanism for the survival of cells under adverse conditions [1]–[3]. Entomopathogenic fungi are microorganisms that infect and typically kill their insect hosts as a developmental necessity. Spores must first overcome chemical and physical barriers of the insect cuticle before responding to the heterogeneous nature of the insect haemocoel, that includes the innate immune system, and metabolizing host materials to fuel growth [4]. Finally, the fungus kills the host before utilizing nutrients from the dead tissue for spore formation and transmission. The multiple steps, involving a variety of stages (cuticle penetration, blastospore growth and vegetative growth) and changing environmental conditions as the parasite encounters different parts of the host, implies significant cellular variability during the different stages of the infection process.

The genera *Beauveria* and *Metarhizium* are both comprised of anamorph entomopathogenic fungi that infect a wide range of arthropod hosts. Fungal strains for both these genera are frequently isolated from soil and have been the most widely employed as mycoinsecticides for the biological control of insect pests worldwide. In recent years, interest in the nature of the secondary metabolites responsible for their pathogenesis has increased. As a result, toxic cyclic depsipeptides, such as beauverolides in *Beauveria* sp. [5]–[8] and destruxins in *Metarhizium* sp. [9]–[11], have been well described, among other secondary metabolites such as beauvericin [12], bassianolide [13] and NG-291 [14]. The similar biological control function of these two entomopathogens against hundreds of species of insects has resulted in a commonly held view that they are ecologically similar species with comparable modes of action leading to similar results [15]–[18]. However, *Beauveria* and *Metarhizium* are phylogenetically distinct by at least 150 million years [19], and some of the different groups of metabolites they produce are described implying different modes of action inside the insect host. Therefore, in spite of their similar effects on tested insect pests, they likely possess significant differences in both the range of metabolites and the manner in which they are deployed.

To address this hypothesis, we adopted a global metabolomics approach in combination with a newly developed *ex vivo* insect culturing assay. As such, we have grown vegetative mycelium of a *Beauveria* and *Metarhizium* isolate in the presence of *Camponotus pennsylvanicus* ant brains and muscles kept alive in an insect cell culture medium. Liquid chromatography coupled with quadrupole time-of-flight mass spectrometry on the medium after prolonged contact between fungus and ant tissue shows that these two fungal isolates indeed secrete a very different array of metabolites (hereafter referred to as their secretome) on both live and dead ant material. In addition, they each exhibit a significantly different secretome when encountering different insect tissues. Furthermore, using this novel culturing approach, we discovered additional cyclic depsipeptides for both species that have not yet been described. These compounds have been reported to have roles in cellular paralysis and cytotoxic effects, which have been shown to have an antitumor, antibiotic, antifungal, immunosuppressant and anti-inflammatory effect [20]–[24]. Finally, we discovered that these insecticidal compounds are heterogeneously secreted depending on the type of tissue: the *Beauveria* isolate secretes its beauverolides mainly on live tissues, while the *Metarhizium* isolate adopts a strategy of releasing destruxins predominantly on dead tissue.

## Results and Discussion

### Analysis of the Heterogeneous Nature of *Metarhizium brunneum* (F52) and *Beauveria bassiana* (GHA)

In this study we made use of a fungal isolate from the species *Metarhizium brunneum* and *Beauveria bassiana*. Their secondary metabolite secretion was tested when vegetative mycelium encountered *C. pennsylvanicus* tissues. Carpenter ants of the species *C. pennsylvanicus* are considered a pest in the United States since they preferably nest in dead wood and cause structural damage to many houses every year [25]. For this experiment we dissected brains and muscles out of heads of *C. pennsylvanicus* workers, something that is commonly being performed in insect physiology and behavioral studies [26]. We chose brain and muscle tissue as fat, malphigian tubule and gut tissue were liable to break extensively during dissections making it unsuitable. Ant brains and muscles were placed in cell culture inserts (Millipore) in Schneider’s medium (Gibco), designed to resemble hemolymph for insect cell culture when freshly supplemented with fetal bovine serum (PAA laboratories Inc.) [27]. As such, *ex vivo* tissues stay viable (though we cannot with certainty state they are metabolically similarly active), creating fungal growth conditions that can be used to simulate an ant infection and examine the reaction of entomopathogens to the different tissues it encounters. Tissue viability was checked after a week of incubation using a trypan blue staining (Sigma), which indicated that ant brain and muscle tissue cultured *ex vivo* were indeed still alive (Figure S1), however similarly tested gut and fat tissue were not. The possibility for using actual hemolymph extracted from live ants as a medium was considered, but rejected because of potential microbial activities. After a 3-day incubation of Schneider’s medium, ant brain and muscle tissue, with and without fungal growth, the supernatant from the different conditions was harvested and prepared for metabolomic analysis.

**Figure 1:**
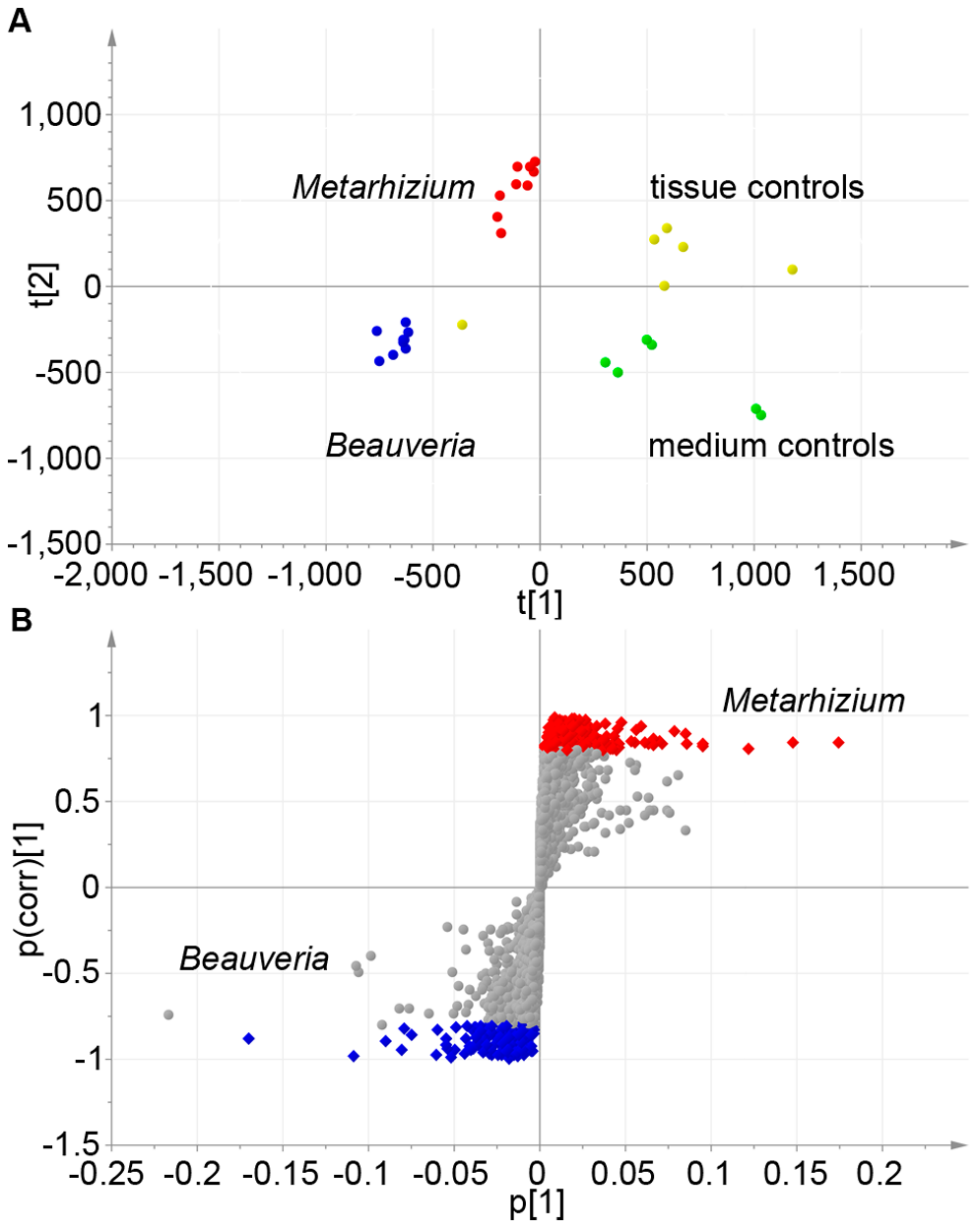
Separate clustering of fungal and control samples used in this study. A) Principle Component Analysis (PCA) of samples incubated in Schneider’s medium supplemented with 10% FBS. Clustering of different sample types based on all samples for medium controls (green), ant brain and muscle controls (yellow), *Beauveria* growth (blue), and *Metarhizium* growth (red) in a three-dimensional score space. B) S-plot analysis of all *Metarhizium* samples against all *Beauveria* samples. All *Metarhizium* related ions with p(corr) [1]>0.8 are marked red, while all *Beauveria* related ions with p(corr) [1]>0.8 are marked blue.

Biological triplicates of the different samples were run in a randomized order using an AB Sciex (Framinhgam, MA) 5600 quadrupole time-of-flight mass spectrometer connected to a Shimadzu (Columbia, MD) UFLC system. Prior to data analysis the raw LC-MS peak data of all 60 samples were aligned together using the parameters described in the material and methods. Subsequently, the dataset was processed to include only monoisotopic ions between 100 and 800 m/z with retention times between 1 and 16 minutes. This resulted in a total of 15000 unique retention time/mass to charge ratio peaks (Data File S1, http://www.metabolomics.psu.edu) on which extensive analyses, using both univariate and multivariate statistics, were performed. Principal component analysis (PCA) showed that despite the complex nature of the medium used, there is a distinguishable clustering of the four major groups of samples: medium controls, ant tissue controls, *Beauveria* growth and *Metarhizium* growth (Figure 1a). This clustering allows us to confidently claim that although we are using complex media and tissue conditions, due to the incorporation of controls for the medium, ant tissues and fungal growth in the medium, we can distinguish fungal metabolites from those found in the cell culture media and the insect tissues. In addition to demonstrating the ability to conduct global metabolomics profiling on *ex vivo* tissues, this approach revealed the differences in secretomes between isolates of these two generalist fungal species since they secreted a significantly different array of metabolites, when experiencing to the same environment. Comparing by t-test all ions secreted by the *Beauveria* isolate to those of the *Metarhizium* isolate, 2540 and 1177 ion peaks respectively, were found to be secreted in significantly different amounts (p<0.01) and of those 31.5% and 32.2% were unique to either of the fungal isolates (Data File S2, sheet 1 and 2). To illustrate this the loading data for 10 representative peaks found using this analysis can be found in Figure S2a,b. A similar comparison was done making use of the OPLS-DA (orthogonal projections to latent structures discriminant analysis) and S-plot analysis using SIMCA-P+13. This multivariate analysis facilitates the identification of potentially biochemically significant metabolites since the S-plot reveals which ions are driving the clustering of the groups being compared. Using a cut off of p(corr) [1]>0.8 (Figure 1b) this analysis identified 372 and 328 significantly different retention time/mass to charge ratio signals for the *Beauveria* and *Metarhizium* isolates respectively (Data File S3, sheet 1). Given their different and distant position on the phylogenetic tree of the order Hypocreales [28], [29] different secretomes are expected. This expectation is strengthened by a recent comparative genomics study in which *B. bassiana* was compared to two *Metarhizium* species as well as *Cordyceps militaris* [30]. The study revealed the differences and similarities of gene clusters encoding for e.g. toxins, enzymes and secondary metabolites in these fungi and demonstrated that insect pathogenicity in *B. bassiana* and *C. militaris* evolved independently from *Metarhizium* [30]. Our metabolomic analyses shows how this phylogenetic independence results in a significantly different array of secreted secondary metabolites. Since both of these fungi are used in the biological control of insect pests, understanding the nature of their heterogeneous secretomes could help in designing more effective control measures.

**Figure 2:**
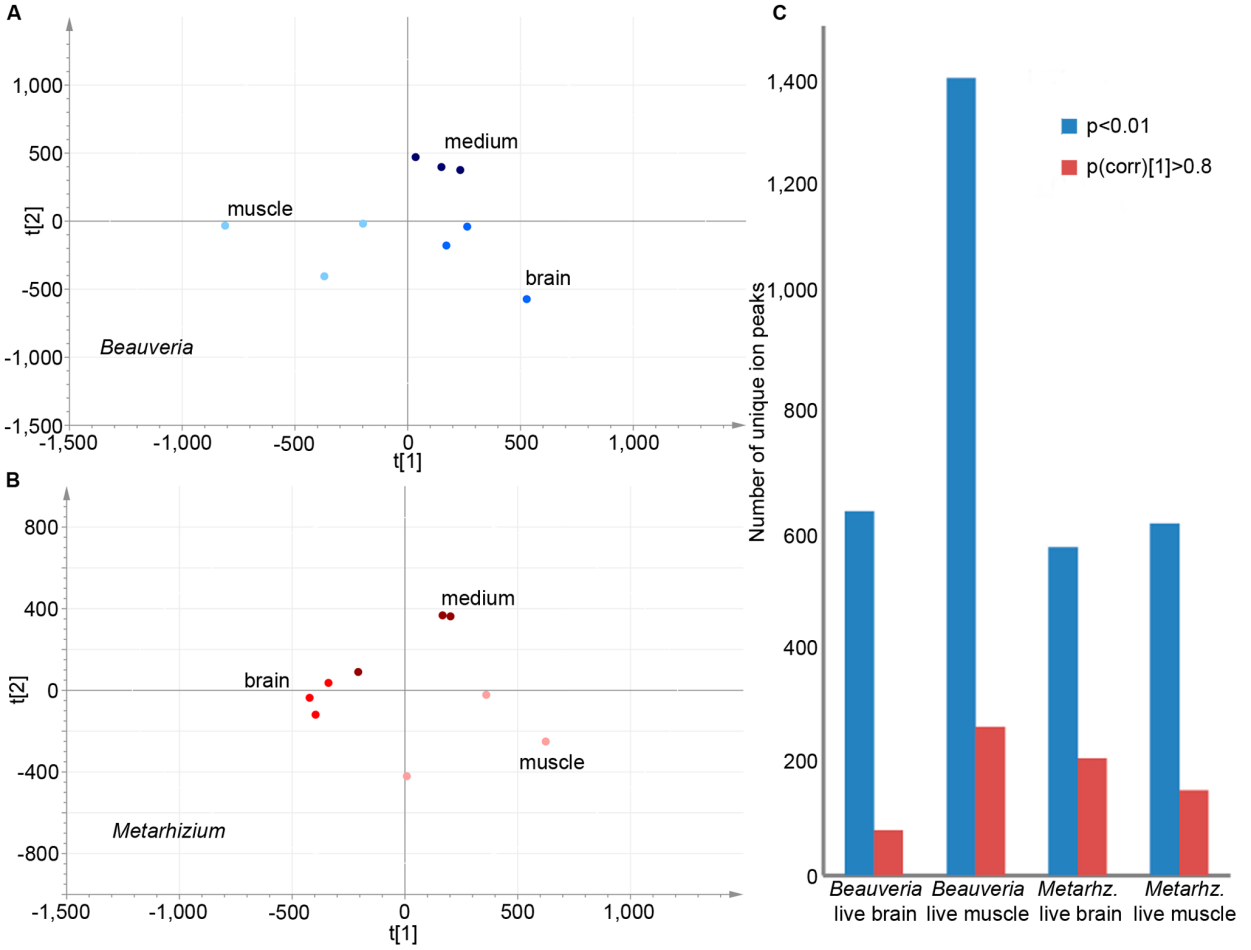
Overview of analyses to determine the tissue specificity of *Beauveria* and *Metarhizium*. A) Supervised PCA plot showing the clustering of different types of *Beauveria* growth; growth on Schneider’s medium (dark blue), Schneider’s medium with ant brains (bright blue) and Schneider’s medium with ant muscles (light blue). B) Supervised PCA plot showing the clustering of different types of *Metarhizium* growth: growth on Schneider’s medium (dark red), Schneider’s medium with ant brains (bright red) and Schneider’s medium with ant muscles (light red). C) Bar graph displaying the number of unique ion peaks found to be significantly specific for growth on either live brain or muscle tissue. Both statistical methods used in this study are represented, p<0.01 being the number of peaks found significant from the t-test and p(corr) [1]>0.8 the ones found significant for the S-plot analysis.

In addition to having different secretomes these two fungi exhibited contrasting secondary metabolite deployment behavior, demonstrating that, when studying these organisms, we should appreciate the differences underlying their pathogenicity. Distribution of ions in a three-dimensional metabolic space of all growth condition samples within a species resulted for both in a separate supervised clustering for growth on brain tissue, muscle tissue and Schneider’s medium (Figure 2a and b). Our interpretation of these results is that both fungi condition a significant amount of their overall metabolite profile to the two *ex vivo* and control conditions tested, implying they exhibit a heterogeneous approach to encountering different situations and environments within the body of the host. When assessing what signals are significantly different in the various growth conditions, univariate analyses revealed for *Beauveria* 643 and 1349 significant (p<0.01) retention time/mass to charge ratio peaks for growth on live brain and muscle tissue respectively (Figure 2c; Data File S2, sheet 3 and 4; Figure S2c,d). Focusing only on the peaks best fitting the multivariate model (p(corr) [1]>0.8), 75 and 245 signals were found on live brain and muscle tissue respectively (Figure 2c; Data File S3, sheet 2 and 3). This indicates that the *Beauveria* isolate specifically increases the secretion of 2.1–3.3 times more compounds when in contact with live ant muscle tissue compared to growth on live ant brain tissue. For *Metarhizium,* we also found a significant amount of tissue growth dependent ions. However, in contrast to the *Beauveria* isolate, similar specificity numbers were found for brain and muscle tissue growth (Figure 2c; Data File S2, sheet 5 and 6; Figure S2e,f; Data File S3, sheet 4 and 5).

### Fungal Metabolome on Dead Compared to Live Insect Tissues

**Figure 3:**
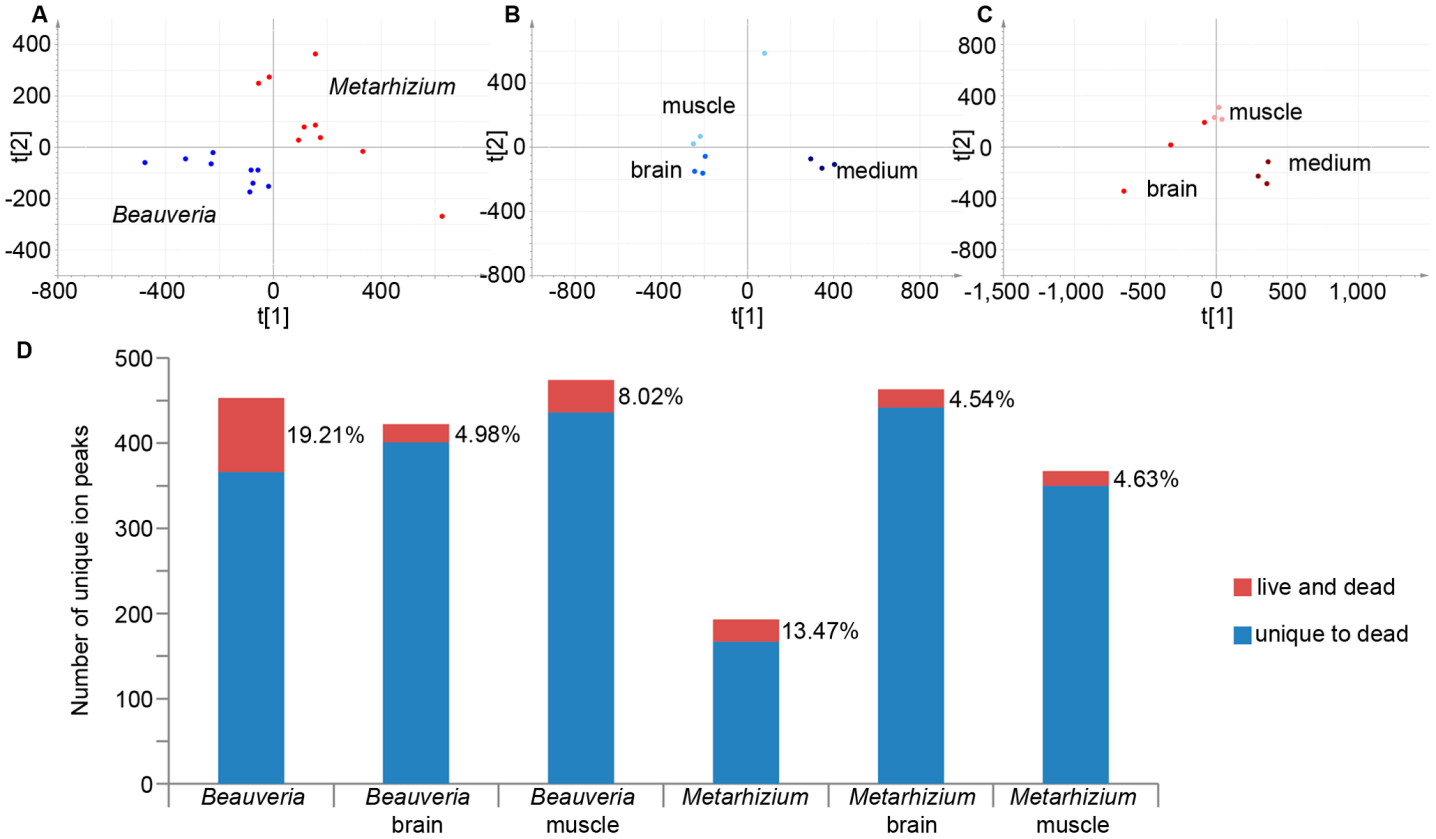
Overview of data obtained from analyses done to determine secretome heterogeneity on dead ant tissues. A-C depict the supervised PCA plots for A) general *Beauveria* and *Metarhizium* growth on dead tissues, B) different types of *Beauveria* growth, and C) different types of *Metarhizium* growth. D) Bar graph representing the number of unique ion peaks found in the comparisons of the two fungal species with each other and the comparison of dead tissue growth related ion peaks within those species. The top part of the bars represents the percentage of metabolites in common with fungal growth on live tissues.

After invasively growing into the insect hemocoel, overcoming the immune system, replicating, and eventually killing the host, entomopathogenic fungi switch their single cell growth to a filamentous one by forming hyphae. The latter uses the insect body as a carbon source for the formation of spore structures for fungal dispersal. Since host death is a developmental necessity for the transmission of this pathogen we can expect that these fungi can respond to cues signaling the death of the host and respond with different secreted compounds. To test this we grew *Metarhizium* and *Beauveria* on *C. pennsylvanicus* tissues kept in PBS. Trypan blue staining 16 hours after dissection showed that ant tissues were dead after a prolonged period in this saline buffer, and fungal hyphae were not able to grow in it unless a carbon source was added (Figure S1). PCA revealed that these PBS grown samples cluster separately from the samples discussed above. This is to be expected since PBS and the Schneider’s medium used above consist of very different compounds. Also, these fungi did not grow in PBS, which resulted in completely different background LC-MS signals and less generated ion peaks overall. A direct comparison between our live tissue data set and the dead tissue dataset is thus not valid. However, we can analyze the dead tissue data set separately, using the same bioinformatics tools we employed above, and make the comparison based on the output afterwards. OPLS-DA is used in all cases for this subset of the data since there were not enough abundant ion peaks that were significantly different to cluster the different sample types using PCA. This again resulted in data suggesting the different metabolic nature of *Beauveria* versus *Metarhizium* (Figure 3a) and the heterogeneous reaction to different insect tissues of both fungi (Figure 3b and c). Again a great amount of significantly different signals for the different comparisons made were found, which are all again taken up in Data File S2 (sheets 7–12) and Data File S3 (sheets 6–10). The graph in Figure 3d presents an overview of all the significant (p<0.01) monoisotopic ion peaks found using univariate analysis. Only 4.54–19.21% of these peaks were also found when these fungi were grown on live ant material. Metabolomics on these two experimental set ups thus demonstrate that entomopathogenic fungi can distinguish between dead and live host material and as such exhibit a different metabolic response. This strengthens our point that, in order to study secondary metabolite secretion in entomopathogens, the heterogeneous nature of fungal entomopathogens should be taken into account when designing a culturing method.

### Discovery of Novel Cyclic Depsipeptides

Metabolomics coupled with *ex vivo* organ culturing has allowed us to begin the important process of understanding the key differences between two ecologically and agronomically important fungal isolates. It also allowed us to create an experimental environment in which we simplified host complexity in ways that will bring us closer to the understanding of the heterogeneous mechanisms underlying insect infection compared to other culturing systems reported for metabolite discovery in this important group of fungi [31]. This created a significant opportunity for novel metabolite discovery, which is of general importance given the increasing prominence of these fungi as bio control agents. However, a possible roadblock is that public metabolite databases do not yet hold an extensive amount of information, making it not yet possible to identify metabolites by automated library searching.

**Figure 4:**
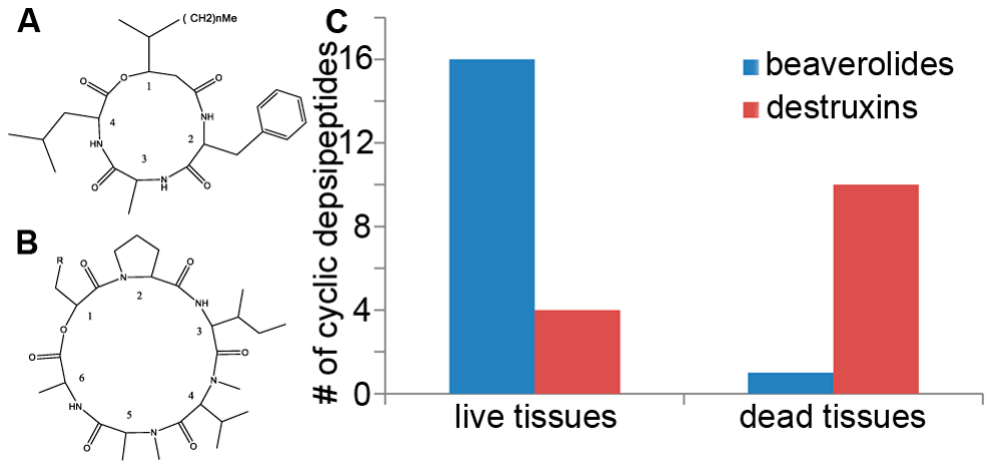
Overview cyclic depsipeptides identified in this study. A) General structure of beauverolides. The identities of the amino acids at positions 2–4 and the n descriptor are given in Table 1. B) General structure of destruxins. The identities of the amino acids at positions 2–6 and the R descriptor are given in Table 2. C) Bar chart summarizing the amount of beauverolides (blue) and destruxins (red) found to be secreted on live and dead insect tissues.

For both *Beauveria* and *Metarhizium* the cyclic depsipeptides, that are found to play a role in pathogenesis, are well studied. For both cyclic depsipeptides several derivatives have been identified that exhibit different rates of activity in the conditions tested to study their effects. Beauverolides secreted by *Beauveria* sp. are cyclic tetradepsipeptides containing C_9_- or C_11_-β-hydroxy acid residues [32] (Figure 4a). More than twenty different beauverolides have been identified, and sequencing of beauverolides by tandem mass spectrometry has been well described [32], [33]. The product ion mass spectra we obtained in this study were virtually identical to those in the literature. The presence of an immonium ion at m/z 139 or m/z 167 indicates the presence of hydroxy-methyloctanoyl and hydroxy-methyldecanoyl residues respectively at position 1. The presence of additional immonium ions at m/z 72, 86, 104, 120, 139 and 157 indicates the presence of certain amino acids, valine, leucine and isoleucine, methionine, phenylalanine, tyrosine and tryptophan respectively. The presence of prominent b_3_, b_2_-H_2_O, a_2_-H_2_O, and 3–4 dipeptide ions allow for the complete sequencing of these compounds. Destruxins secreted by *Metarhizium* sp. are cyclic hexadepsipeptides consisting of an α-hydroxy acid and 5 amino acid residues (Figure 4b). Thirty-nine different destruxins have been reported [10]. The mass spectral fragmentation patterns of destruxins have also been well characterized [34]–[36], and again the product ion mass spectra we obtained in this study were virtually identical to the in-source CID mass spectra previously reported. The D-alpha-hydroxy acid residue, which is a major site of heterogeneity in the destruxins, gives a prominent fragment ion designated D^+^ (34), allowing the identification of this residue. The accurate mass capabilities of the mass spectrometer used in this study easily discriminated between the isobaric 2-hydroxy-4-methylpentanoic and 2-hydroxy-4,5-epoxypentanoic side chains. The presence of prominent b_5_, b_4_, a_4_ b_3_ and b_2_ ions allows the sequence determination of these compounds. Depending on the amino acids available in the environment, derivatives of these secondary metabolites are being formed that can have different activities and biological functions within metabolic pathways.

Analyzing the metabolite peaks obtained in this study we have identified 20 beauverolides of which 14 have not been described before (Table 1) and 14 destruxins of which three are novel (Table 2). The mass spectra for all these cyclic depsipeptides and their predicted structures can be found in Data File S4. As expected, when comparing the metabolome of these two fungi, these cyclic depsipeptides turn up as being species specific (Data File S2, sheets 1,2 and 8). Next to that they seem to be predominantly secreted (p<0.01) as a result of interacting with one of the two tissues with a fold change on brain or muscle tissue ranging between 4.4 and 128.3 (Tables 1 and 2). Comparing this result again to the multivariate analysis by means of S-plot analysis however, only half of the beauverolides and one of the destruxins are found to be above p(corr) [1]>0.8 when comparing all *Beauveria* to all *Metarhizium* samples. Using this statistical analysis to look at tissue specificity, none of them appear to be above the set threshold of 0.8. This implies that although these important secondary metabolites are clearly heterogeneously secreted, they are overall not the main heterogeneous compounds driving PCA clustering of the different sample conditions used in this study. Other, yet unidentified compounds seem to be of more biochemical significance. We therefore hope our supplementary data sets will help researchers interested in the secondary metabolites of these two fungi to identify those compounds eventually.

**Table 1:** Secreted beauverolides in the *ex vivo* culturing system.

[M+H]^+^	RT/min	n	AA2	AA3	AA4	beauverolide	fold change on brain	fold change on muscle
482.3261	13.99	3	Val	Lxx	Lxx	*this study*		L 4.6D ∞
488.2894	13.54	3	Ala	Phe	Lxx	*this study*	L 5.9	
500.3652	13.62	3	Val	Met	Lxx	*this study*		L 4.4
510.3574	15.07	5	Val	Lxx	Lxx	*this study*		L 6.3
514.3808	13.93	3	Lxx	Met	Lxx	*this study*		L 6.2
516.3432	13.98	3	Val	Phe	Lxx	E		L 6.1
528.3965	14.5	5	Val	Met	Lxx	*this study*		L 6.2
530.3588	14.3	3	Lxx	Phe	Lxx	*this study*		L 6.2
532.3426	13.3	3	Val	Tyr	Lxx	*this study*	L 4.7	
544.3745	15.01	5	Val	Phe	Lxx	B		L 7.7
558.3902	15.5	5	Lxx	Phe	Lxx	*this study*		L 8.8
560.3739	14	5	Val	Tyr	Lxx	*this study*		
564.3872	14.24	3	Phe	Phe	Lxx	F		
576.4405	14.74	5	Phe	Met	Lxx	*this study*		
580.3866	13.54	3	Tyr	Phe	Lxx	*this study*		L 4.4
592.4073	15.4	5	Phe	Phe	Lxx	C		L 9.9
603.4239	14.02	3	Trp	Phe	Lxx	J		L 6.8
608.4067	14.28	5	Tyr	Phe	Lxx	*this study*		
615.466	14.58	5	Trp	Met	Lxx	*this study*		L 8.2
631.444	14.99	5	Trp	Phe	Lxx	K		L 8.7

For each identified beauverolide the accurate mass of the protonated molecular ion, retention time, n, and the identities of the amino acids at positions 2–4 are given (Figure 4a). The ones discovered in other studies are indicated by the letter assigned to them. The fold changes on brain and muscle indicate the tissue specificity of beauverolides. All of them are heterogeneously secreted on live tissues (indicated by L), while one is uniquely secreted on dead muscle tissue as well (indicated by D). The beauverolides mentioned in gray were identified in our raw data set but were not of enough abundance to be used in the further data analysis.

**Table 2:** Secreted destruxins in the *ex vivo* culturing system.

[M+H]^+^	RT/min	R	AA2	AA3	AA4	AA5	AA6	destruxin	fold change on brain	fold change on muscle
564.3392	11.53	CH = CH_2_	Pro	Val	mVal	mAla	Ala	A_2_	D 9.7	
564.3392	10.92	CH = CH_2_	Pro	Lxx	Val	mAla	Ala	*DesmA*	L 5.4	
566.3392	11.64	CH-(CH_3_)_2_	Pro	Val	Val	mAla	Ala	*this study*		
578.3548	11.7	CH = CH_2_	Pro	Lxx	mVal	mAla	Ala	A	D 24.7	
580.3340	9.34	CH(O)CH_2_	Pro	Lxx	Val	mAla	Ala	*this study*	D 128.3	
580.3704	12.22	CH-(CH_3_)_2_	Pro	Val	mVal	mAla	Ala	B_2_	D 13.5	
580.3704	12.5	CH-(CH_3_)_2_	Pro	Lxx	Val	mAla	Ala	*DesmB*	D 9.0	
594.3861	12.73	CH-(CH_3_)_2_	Pro	Lxx	mVal	mAla	Ala	B	D 36.2	L 23.1
594.3497	10.17	CH(O)CH_2_	Pro	Lxx	mVal	mAla	Ala	E		
596.3654	9.55	COH-(CH_3_)_2_	Pro	Lxx	Val	mAla	Ala	DesmC		
610.3810	10.39	COH-(CH_3_)_2_	Pro	Lxx	mVal	mAla	Ala	C	D 16.3	
610.3446	9.53	CH_2_-CH_2_COOH	Pro	Lxx	Val	mAla	Ala	*this study*		
612.3603	8.69	CH_2_OH-CH_2_OH	Pro	Lxx	mVal	mAla	Ala	E_d_		
624.3603	10.34	CH_2_-CH_2_COOH	Pro	Lxx	mVal	mAla	Ala	D		

For each identified destruxin the accurate mass of the protonated molecular ion, retention time, R and the identities of the amino acids in positions 2–6 are given (Figure 4b). The ones discovered in other studies are indicated by the letter assigned to them. The fold changes found for the majority of them on brain and muscle indicate the tissue specificity of these destruxins. Most of them are heterogeneously secreted on dead tissues (indicated by D), while two are secreted on live tissue (indicated by L). The destruxin mentioned in gray was identified in our raw data set but not of enough abundance to be used in the further analysis of the data.

Lastly, we found that while *Beauveria* secreted its beauverolides mainly in the presence of live muscle tissue, *Metarhizium* secreted its destruxins mostly when in contact with dead brain tissue (Figure 4c, Tables 1 and 2). The latter was unexpected since cyclic depsipeptides are functionally described as “killing” compounds, which is reflected in their name, destruxins. However, our experiment shows that while the *Beauveria* isolate likely uses beauverolides to attack living tissues, the *Metarhizium* isolate secretes destruxins mainly when tissues are already dead. This is not only surprising, but again suggests that the different metabolic approaches these fungi deploy in different environmental circumstances within the host deserve to be studied in more detail.

In conclusion, our data suggests a wealth of difference between two important fungal isolates that are commercially being used in a battle with pest insect species. Although long known to be phylogenetically different the very similar outcomes in tests for biological pest control using these fungi has resulted in them having been considered largely as one tool. What we have shown, using a novel culturing assay with bioinformatics sorting, is that metabolically these fungi behave very differently on different insect tissues. We suggest that knowledge about these differences and the heterogeneous approach when encountering different insect tissues could be important in designing better control measures. Our evidence of different secretomes in these two fungal isolates from two different genera is in line with a recent study resulting in similar conclusions by looking at the genome level [30]. The more artificial culturing methods generally used to study the production of secondary fungal metabolites are not necessarily leading to a good representation of what is happening in nature and might lead to the misinterpretation of metabolic pathways and functions. Furthermore, it might not allow us to identify the derivatives with the highest specific anti-insecticidal activity. Culturing assays, such as the one we developed, would thus aid in understanding the molecular mechanisms underlying pathogenicity of these fungi in their different insect hosts [31].

## Materials and Methods

### Fungal Strains

Experiments were done with strain F52 of *Metarhizium bunneum*, the fungal isolate used in the commercial product Met52® and *Beauveria bassiana* GHA from Botanigard®. Pure axenic cultures were obtained from Nina Jenkins, The Thomas Lab, Dept of Entomology, PSU, and maintained on PDA plates. Inoculations were done using 1.5 µl of a fresh 10^8^ spores/ml saline tween solution (0.8% NaCl, 0.005% Tween-80). Colonies were grown in a two-dimensional way (sandwich culture) using two perforated polycarbonate membranes [37], only allowing the formation of a vegetative mycelium.

### *Ex vivo* Culturing

Ant tissues (brains and muscles) were dissected in PBS from *Camponotus pennsylvanicus* worker’s heads using a stereomicroscope (Nikon SMZ645) holding C-W10× eyepieces and a zoom range of 0.5× to 5×. Prior to dissection, whole ants were surface sterilized in 100% ethanol on ice for 5 minutes and subsequently rinsed in PBS, again on ice. After dissection, tissues were briefly surface sterilized (5–10 seconds) in 100% ethanol and rinsed in Schneider’s media (Gibco) freshly supplemented with 10% fetal bovine serum (FBS) (PAA Laboratories Inc.), 100 µg/mL kanamycin (Invitrogen), 100 units/mL penicillin and 100 µg streptomycin/mL (Invitrogen) [27]. Brains or muscle tissue from the heads of three ants were placed within a 12 µm pore size cell culture insert (Millipore). Using a laminar flow, the tissues were washed three times and finally placed in 1 mL Schneider’s containing FBS and antibiotics as described above for incubation during 3 days at 28°C. To measure metabolites secreted by the strains used in this study, as a reaction to the ant tissues, a 3×3 mm square cut out of a freshly grown sandwich culture was added per single cell culture insert. The proper controls were incorporated by incubating fungal strains without ant tissues, ant tissues without fungal strains, and the medium (with and without antibiotics) without fungal material or ant tissues. Three biological replicates were done for each condition. A similar set up was used for tissues incubated in PBS. Again, brains or muscle tissue from the heads of three ants were placed within a 12 µm pore size cell culture insert (Millipore). Using a laminar flow, the tissues were washed three times and finally placed in 1 mL PBS containing antibiotics as described above for incubation during 3 days at 28°C. To measure metabolites secreted by the strains used in this study, as a reaction to the dead ant tissues, again a 3×3 mm square cut out of a freshly grown sandwich culture was added per single cell culture insert. The proper controls were incorporated by incubating fungal strains without dead ant tissues in PBS, dead ant tissues without fungal strains, and just PBS (with and without antibiotics) without fungal material or ant tissues. While in the live ant tissue set up both fungal strains managed to grow across the diameter of the well, growth stayed local around the tissues in the set up with PBS. In both set-ups fungal growth was not found to be invasive.

### Dead Live Staining

Viability of the *ex vivo* ant tissues in the experimental set up was tested using Trypan blue (Sigma). Medium was removed by draining the cell culture insert and placing it in a clean well. Subsequently the tissues were washed three times using 1 ml PBS. After washing, tissues were stained with 1 ml of a 10-fold dilution of 0.4% Trypan blue (Sigma). The stain was removed by draining and the tissues were again washed 3 times in PBS. Subsequently, fresh medium was added and the tissues were examined using a dissecting scope (Nikon). When tissues failed to absorb the stain they were considered to be viable [38].

### Metabolomics

In order to measure secreted metabolites, tissues were separated from the medium after incubation by draining the cell culture inserts. The medium was harvested, snap frozen using liquid nitrogen, and stored at −80°C upon processing. Subsequently, 100 µl of the medium was mixed with 1 volume of acetonitrile for protein precipitation prior to LC-MS/MS analysis. Samples were randomized and run using the HPLC-QTOFMS (Shimadzu Prominence UFLC XR and AB Sciex 5600 quadrupole time-of-flight mass spectrometry) platform. Samples (5 ul) were separated on a C18 Column (100×2.1 mm 1.7 um, Waters Acquity BEH) using a gradient elution program with aqueous acetonitrile (3–90%) at a flow rate of 250 ul/min. Positive ion electrospray ionization mass spectra were acquired over the mass range 50–1250 Da in IDA (Information Dependent Acquisition) mode with one 100 ms survey scan and up to twenty 100 ms MS/MS product ion scans per duty cycle. The survey scan LC-MS datasets were aligned together using MarkerView (AB Sciex) software and the following parameters were used to extract the peaks from the raw data: Retention times between 0.00 and 16.00 min.; Subtraction Offset of 10 scans; Subtraction Mult. Factor of 1.3; Noise Treshold of 50; Min. Spectral Peak Width of 15 ppm; Min. RT Peak Width of 3 scans; Retention Time Tolerance of 0.10 min.; Mass Tolerance of 10.0 ppm; 3 required samples; Maximum of 100000 peaks. Analyses were done looking at the monoisotopic ions with an m/z between 100 and 800 and a retention time between 1 and 16 minutes, and making use of the multivariate analysis software package SIMCA-P+13 (Umetrics).

## Supporting Information

Figure S1 **Dead/live staining on** ***ex vivo*** **cultured ant tissues.** Result of the trypan blue staining on ant brains (A) and ant muscle (B) kept in Schneider’s insect resembling medium, Grace’s insect resembling medium and PBS for 3 days at 28°C. Tissues failing to absorb the stain are considered viable. Tissues appear to be most viable in Schneider’s medium, hence our choice to use this medium to perform our live tissue experiments in.
(TIF)

Figure S2 **Loading plots of representative peaks found to be significantly different across sample types.** Each panel in this figure shows an example of what the loading plots across sample types of 10 representative peaks found to be significant in the univariate analyses done in this study, looked like. This figure illustrates the striking differences between and the reproducibility within sample types when comparing A) all *Beauveria* samples to all other samples, B) all *Metarhizium* samples to all other samples, C) *Beauveria* grown on live brain tissue versus other types of *Beauveria* growth, D) *Beauveria* grown on live muscle tissue versus other types of *Beauveria* growth, E) *Metarhizium* grown on live brain tissue versus other types of *Metarhizium* growth, and F) *Metarhizium* grown on live muscle tissue versus other types of *Metarhizium* growth.
(TIF)

Data File S1 **All unique retention time/mass to charge ratio peaks that are monoisotopic and found between 100 and 800 m/z with retention times between 1 and 16 minutes.**
(XLSX)

Data File S2 (XLSX)

Data File S3 (XLSX)

Data File S4 (PDF)
