# Supplementary figures and images for: Metabolomics Reveals the Heterogeneous Secretome of Two Entomopathogenic Fungi to Ex Vivo Cultured Insect Tissues
Source: PLoS One. 2013 Aug 5;8(8):e70609. doi: 10.1371/journal.pone.0070609 (PMC3734240; doi:10.1371/journal.pone.0070609)

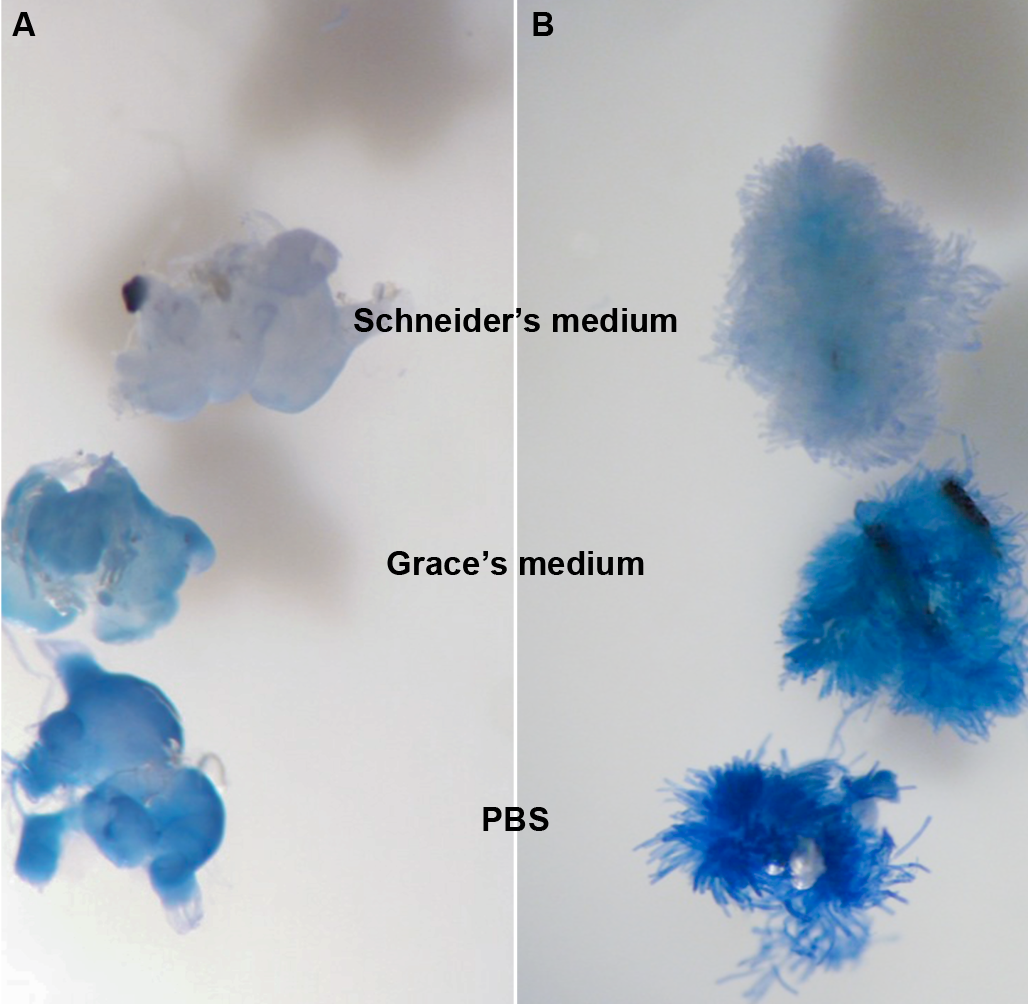

Supplement: Figure S1 — Dead/live staining on ex vivo cultured ant tissues. Result of the trypan blue staining on ant brains (A) and ant muscle (B) kept in Schneider’s insect resembling medium, Grace’s insect resembling medium and PBS for 3 days at 28°C. Tissues failing to absorb the stain are considered viable. Tissues appear to be most viable in Schneider’s medium, hence our choice to use this medium to perform our live tissue experiments in. (TIF) [file pone.0070609.s001.tif]

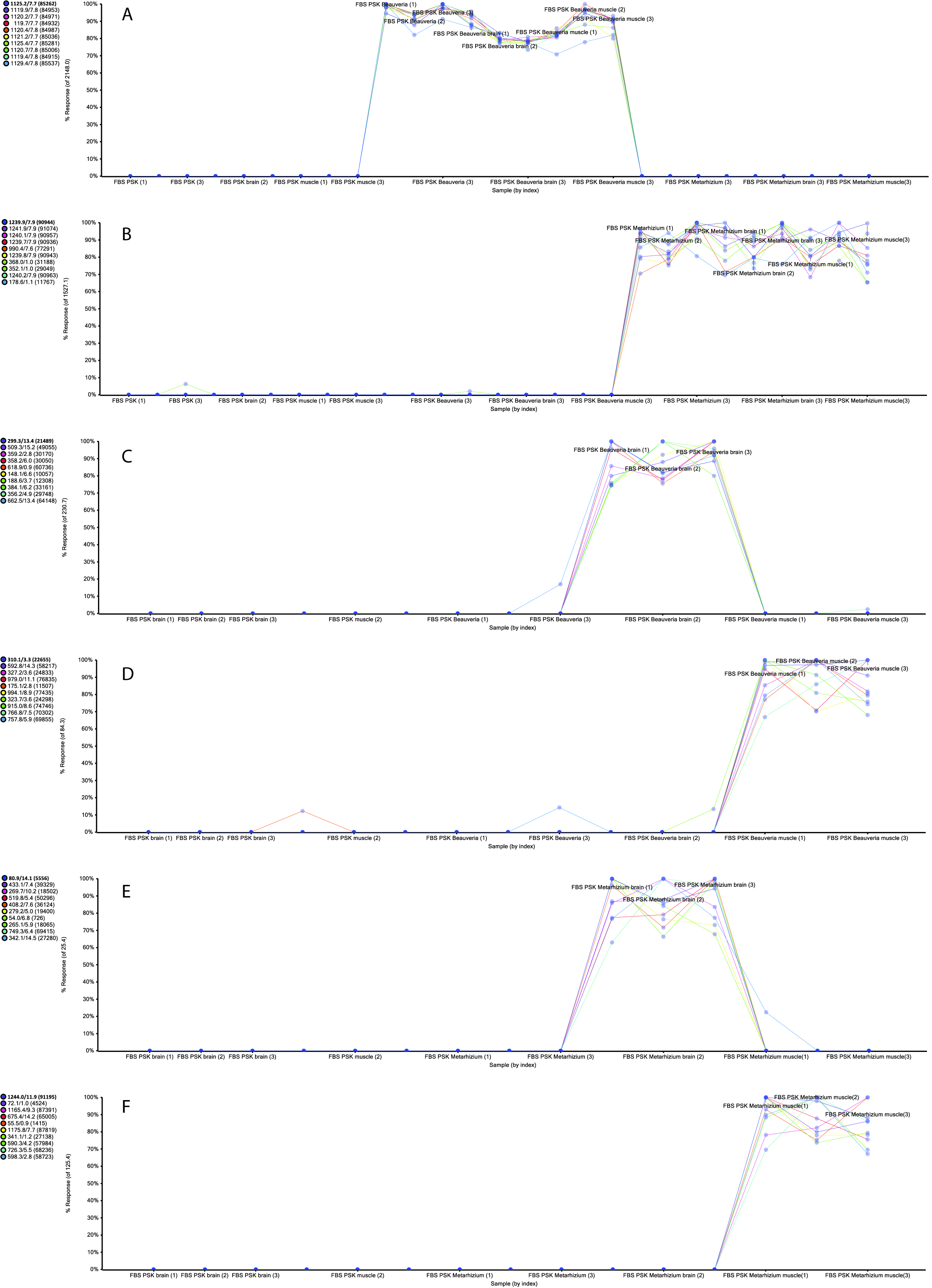

Supplement: Figure S2 — Loading plots of representative peaks found to be significantly different across sample types. Each panel in this figure shows an example of what the loading plots across sample types of 10 representative peaks found to be significant in the univariate analyses done in this study, looked like. This figure illustrates the striking differences between and the reproducibility within sample types when comparing A) all Beauveria samples to all other samples, B) all Metarhizium samples to all other samples, C) Beauveria grown on live brain tissue versus other types of Beauveria growth, D) Beauveria grown on live muscle tissue versus other types of Beauveria growth, E) Metarhizium grown on live brain tissue versus other types of Metarhizium growth, and F) Metarhizium grown on live muscle tissue versus other types of Metarhizium growth. (TIF) [file pone.0070609.s002.tif]
