# Supplementary material for: Metabolomics Reveals the Heterogeneous Secretome of Two Entomopathogenic Fungi to Ex Vivo Cultured Insect Tissues
Source: PLoS One. 2013 Aug 5;8(8):e70609. doi: 10.1371/journal.pone.0070609 (PMC3734240; doi:10.1371/journal.pone.0070609)

Beauverolides

Product ion mass spectra of the 20 beauverolides found in this study in the order as described in Table 1

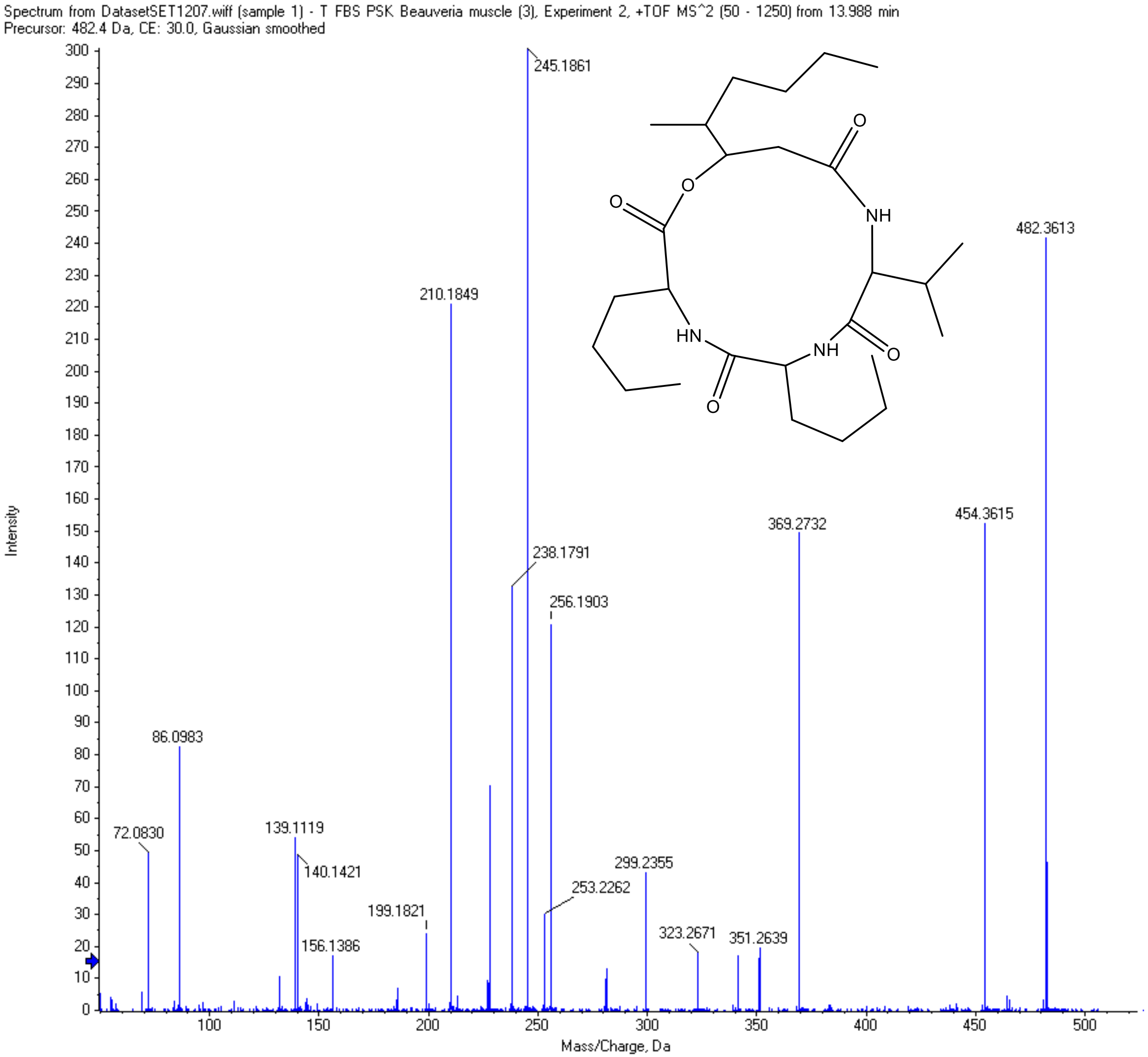
Spectrum from DatasetSET1207.wiff (sample 1) - T FBS PSK Beauveria muscle (3), Experiment 2, +TOF MS^2 (50 - 1250) from 13.988 min
Precursor: 482.4 Da, CE: 30.0, Gaussian smoothed
Intensity
Mass/Charge, Da
245.1861
210.1849
482.3613
454.3615
369.2732
238.1791
256.1903
86.0983
72.0830
139.1119
140.1421
156.1386
199.1821
253.2262
299.2355
323.2671
351.2639
O
NH
HN
NH

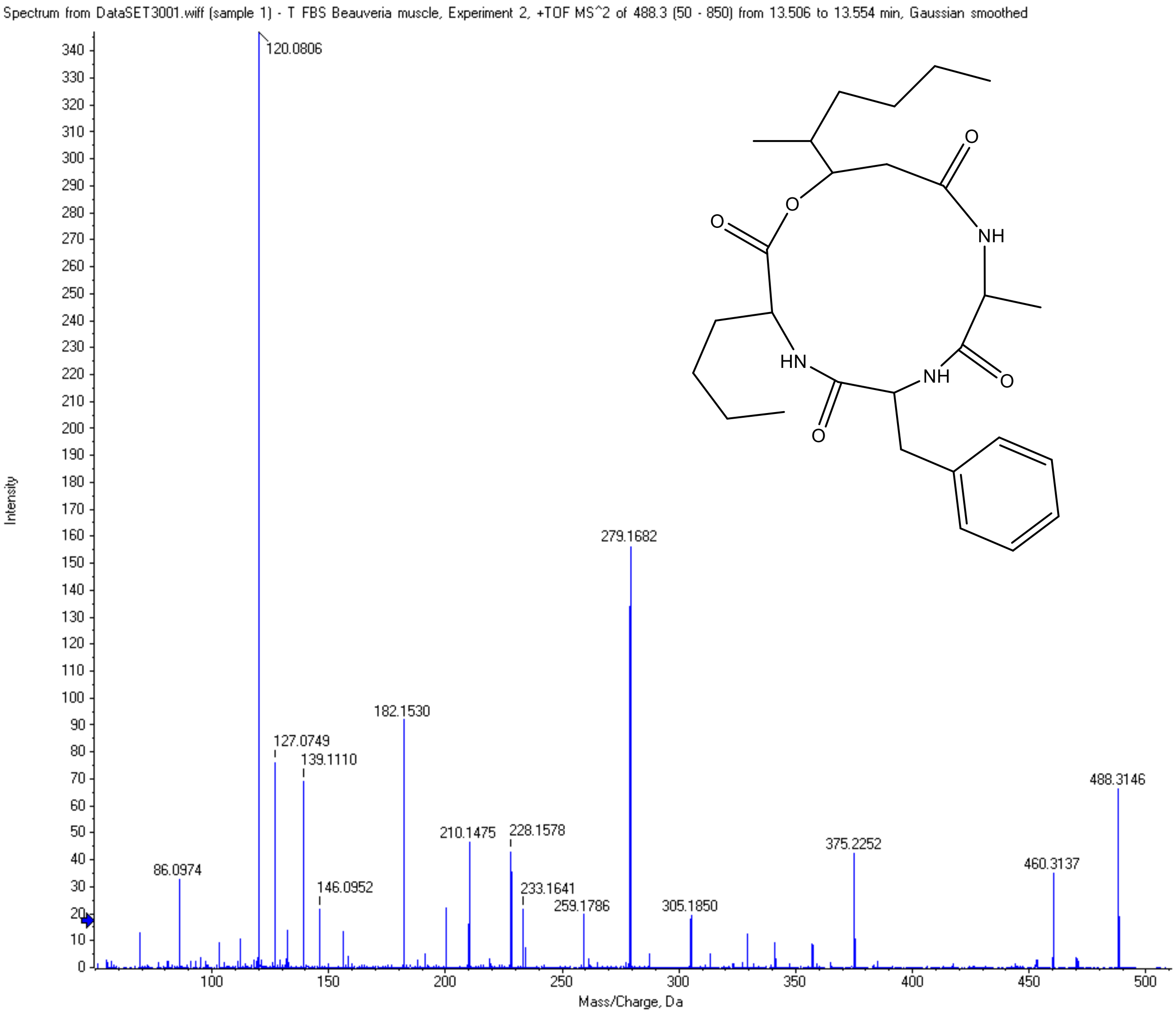
Spectrum from DataSET3001.wiff (sample 1) - T FBS Beauveria muscle, Experiment 2, +TOF MS^2 of 488.3 (50 - 850) from 13.506 to 13.554 min, Gaussian smoothed
Intensity
Mass/Charge, Da
120.0806
279.1682
182.1530
127.0749
139.1110
488.3146
210.1475
228.1578
375.2252
460.3137
233.1641
86.0974
146.0952
259.1786
305.1850
O
NH
HN
NH

Spectrum from DatasetSET1207.wiff (sample 1) - T FBS PSK Beauveria muscle (3), Experiment 3, +TOF MS^2 (50 - 1250) from 13.611 min
Precursor: 500.3 Da, CE: 30.0
With 1 other merged spectrum, Gaussian smoothed

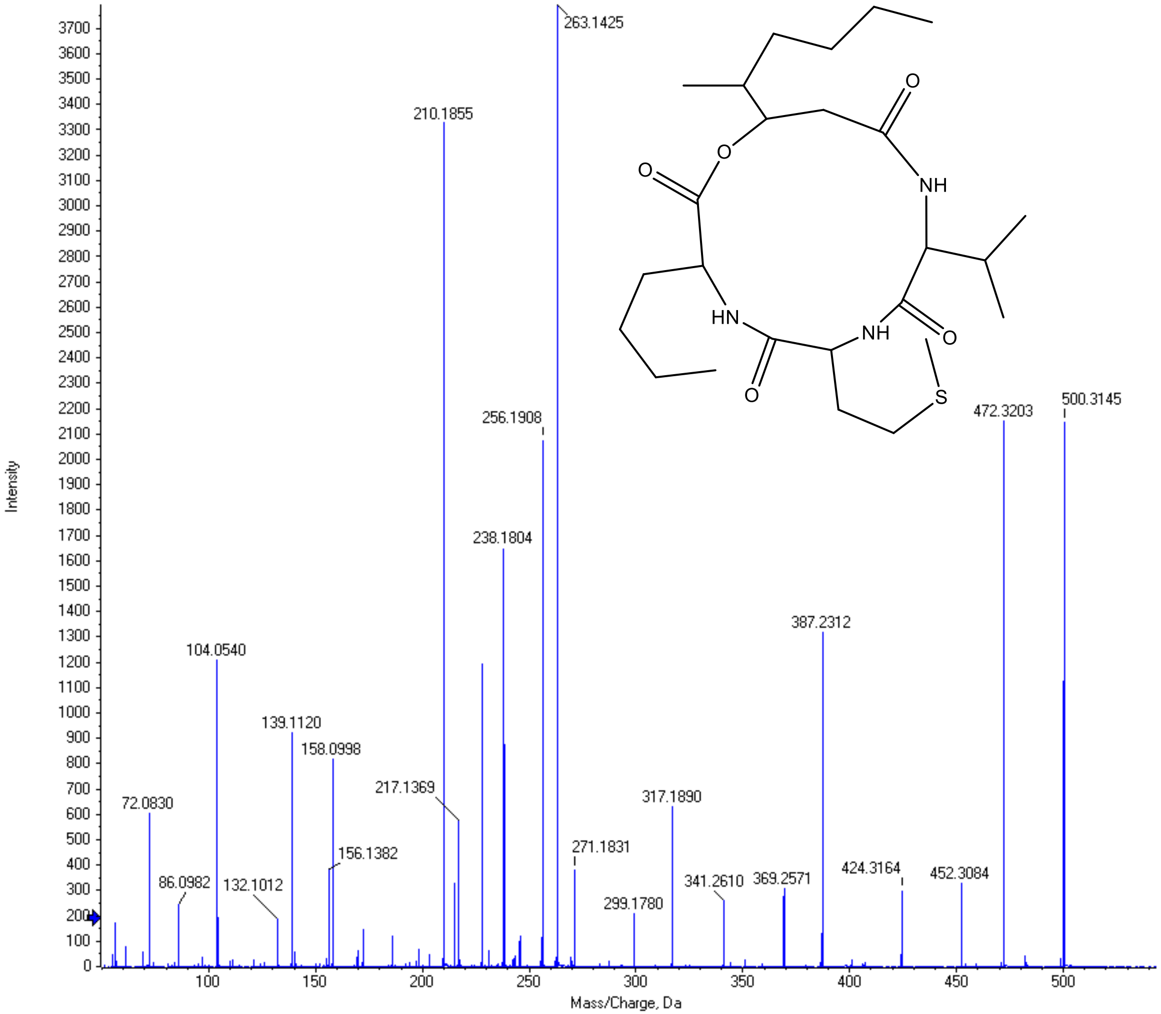

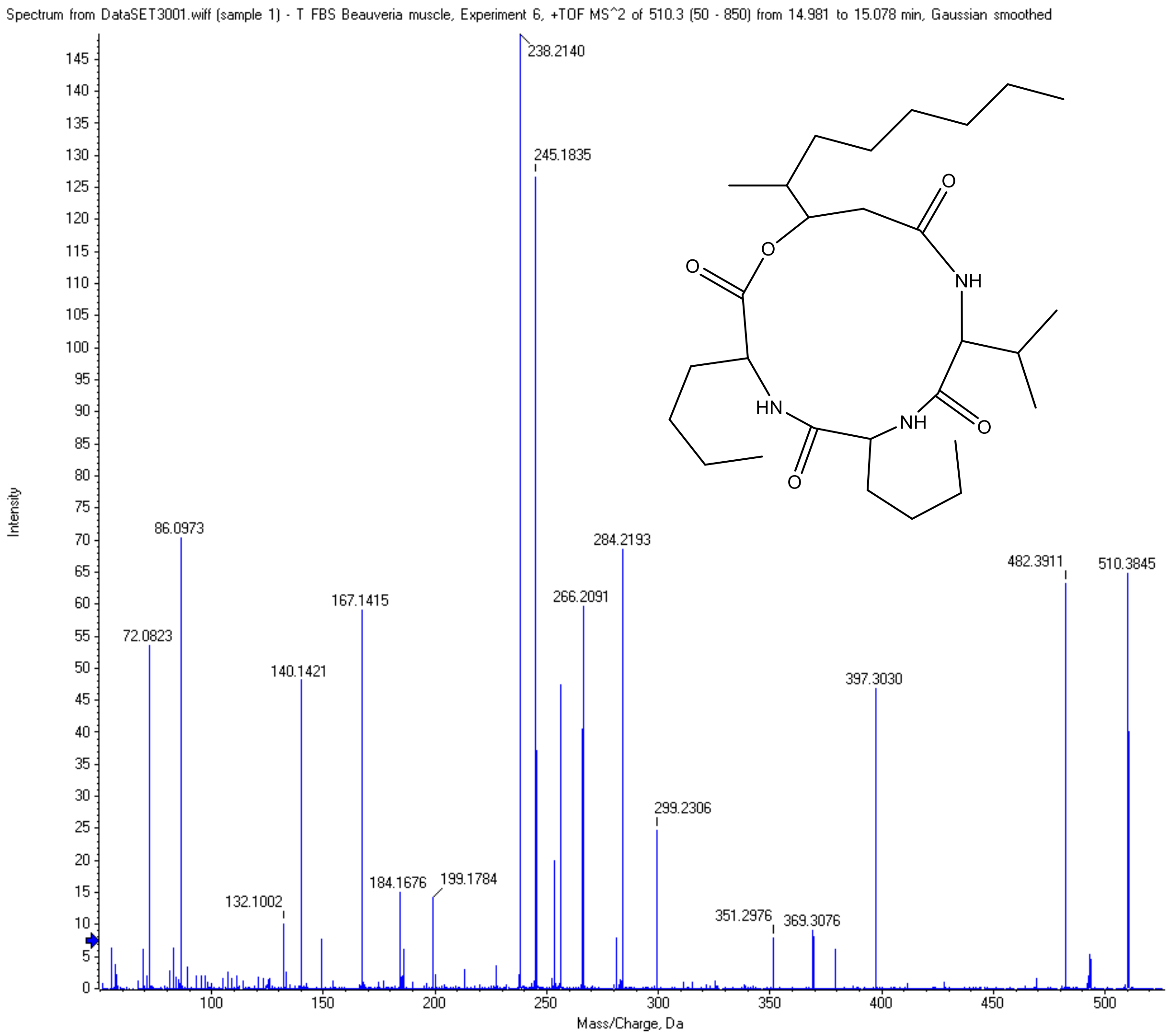
Spectrum from DataSET3001.wiff (sample 1) - T FBS Beauveria muscle, Experiment 6, +TOF MS^2 of 510.3 (50 - 850) from 14.981 to 15.078 min, Gaussian smoothed
Intensity
Mass/Charge, Da
238.2140
245.1835
86.0973
72.0823
140.1421
167.1415
132.1002
184.1676
199.1784
266.2091
284.2193
299.2306
351.2976
369.3076
397.3030
482.3911
510.3845
O
NH
HN
NH

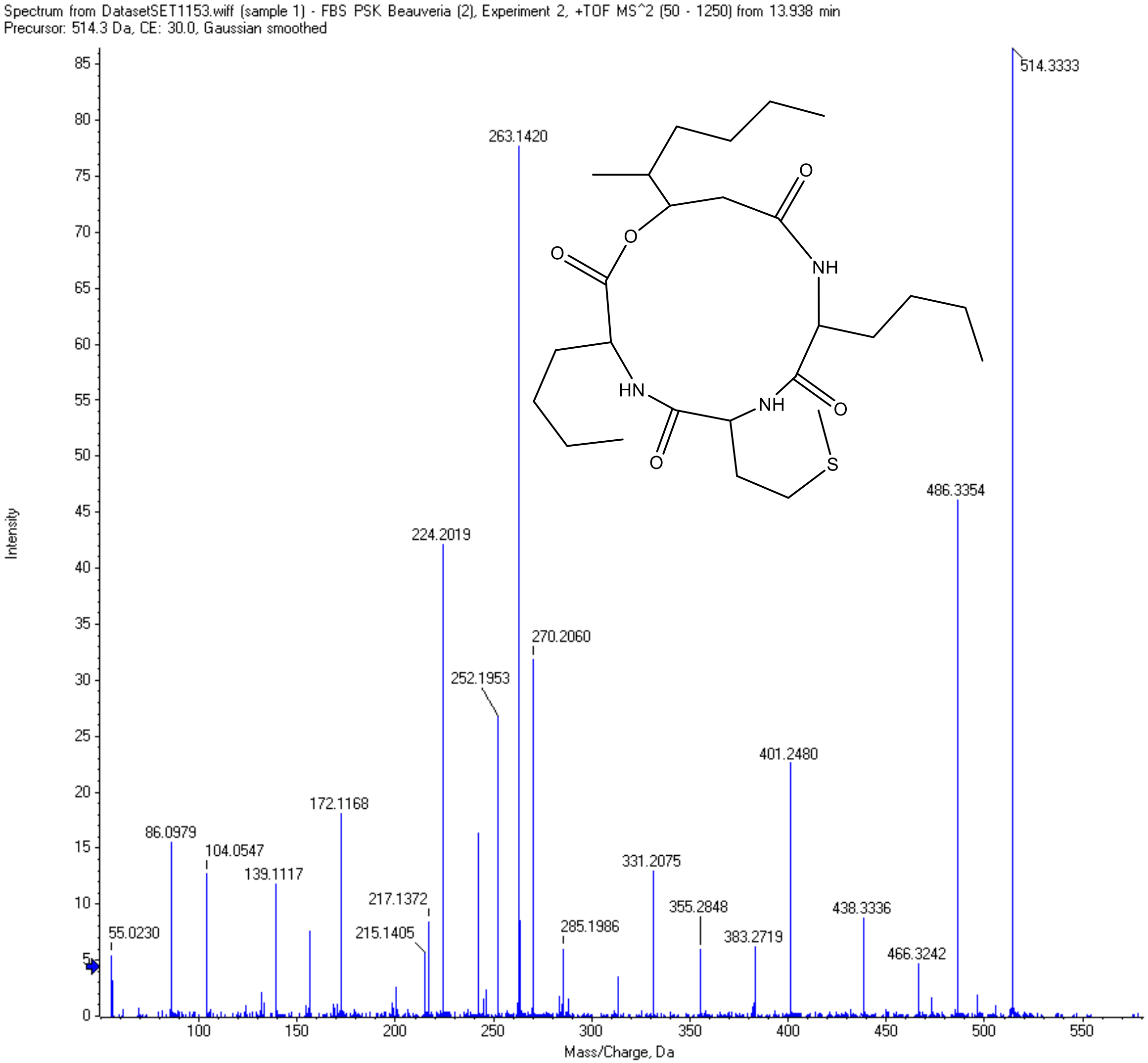

Spectrum from DatasetSET1153.wiff (sample 1) - FBS PSK Beauveria (2), Experiment 2, +TOF MS^2 (50 - 1250) from 13.938 min
Precursor: 514.3 Da, CE: 30.0, Gaussian smoothed
Intensity
Mass/Charge, Da
514.3333
263.1420
486.3354
224.2019
270.2060
252.1953
401.2480
172.1168
86.0979
104.0547
139.1117
331.2075
217.1372
215.1405
355.2848
438.3336
285.1986
383.2719
466.3242
55.0230
O
NH
HN
NH
S

Spectrum from DatasetSET1196.wiff (sample 1) - T FBS PSK Beauveria (1), Experiment 2, +TOF MS^2 (50 - 1250) from 14.027 min
Precursor: 516.3 Da, CE: 30.0
With 1 other merged spectrum, Gaussian smoothed

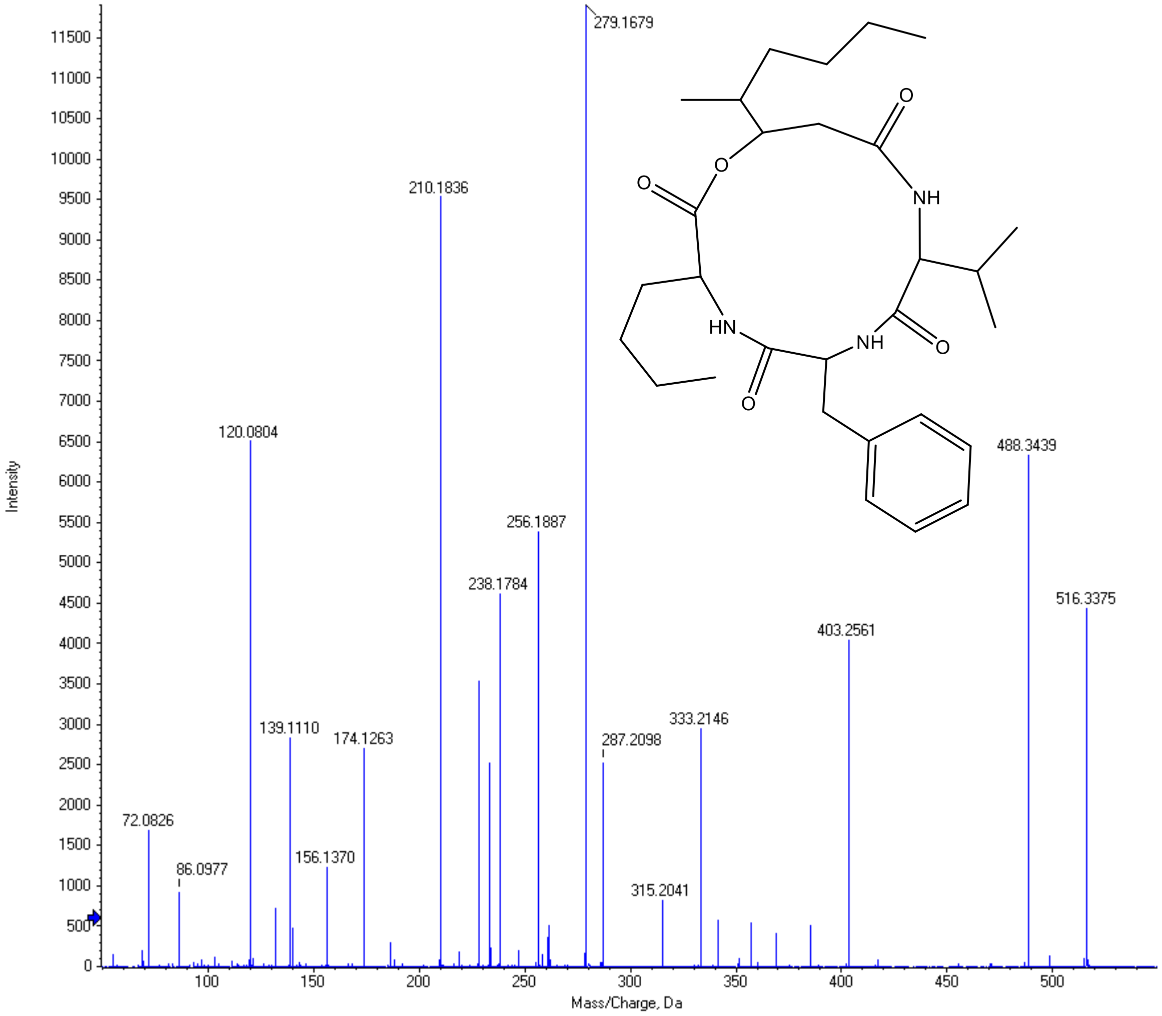

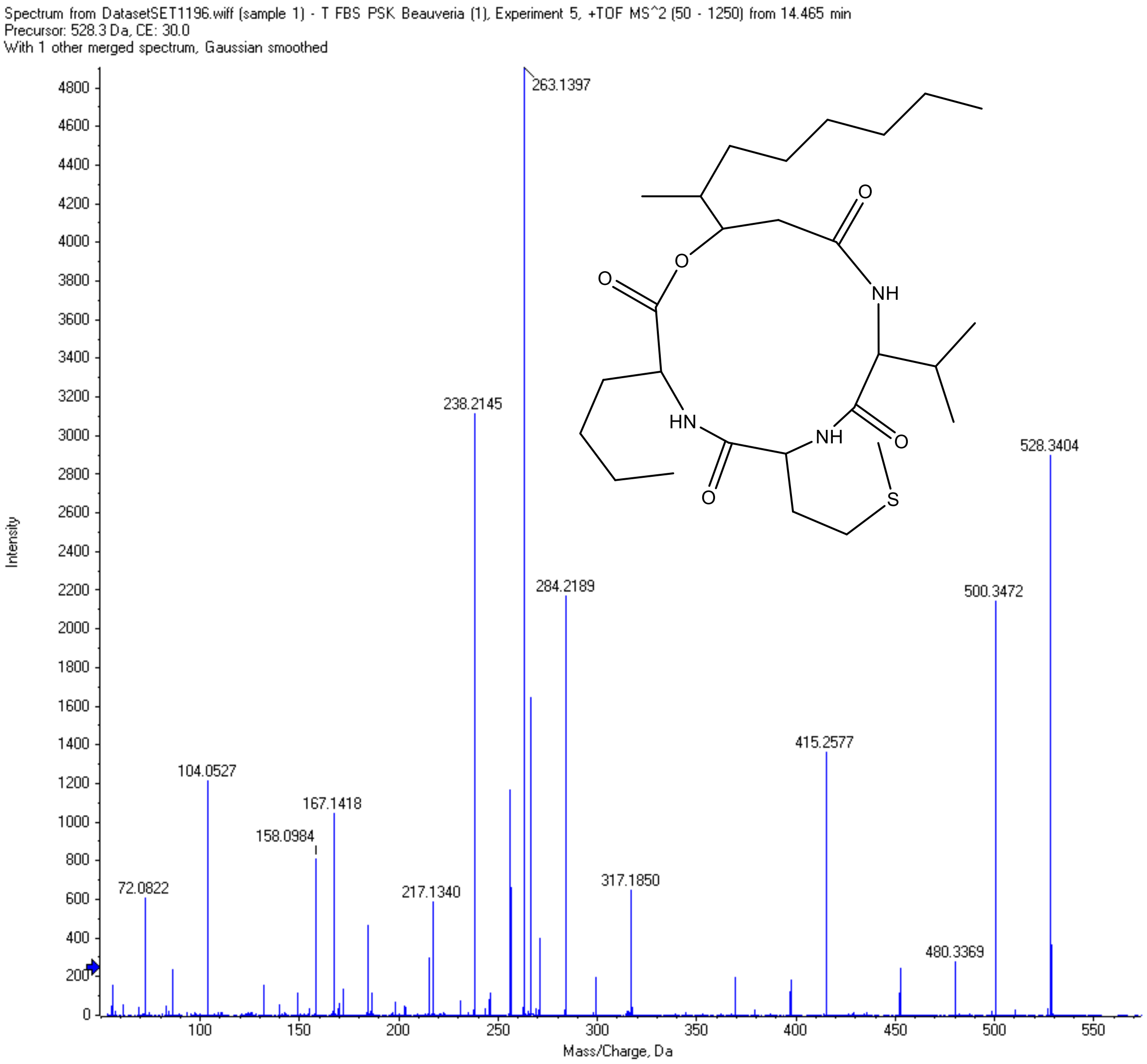

Spectrum from DatasetSET1196.wiff (sample 1) - T FBS PSK Beauveria (1), Experiment 5, +TOF MS^2 (50 - 1250) from 14.465 min
Precursor: 528.3 Da, CE: 30.0
With 1 other merged spectrum, Gaussian smoothed
263.1397
238.2145
528.3404
284.2189
500.3472
415.2577
104.0527
167.1418
158.0984
317.1850
217.1340
72.0822
480.3369
Intensity
Mass/Charge, Da
O
NH
HN
S

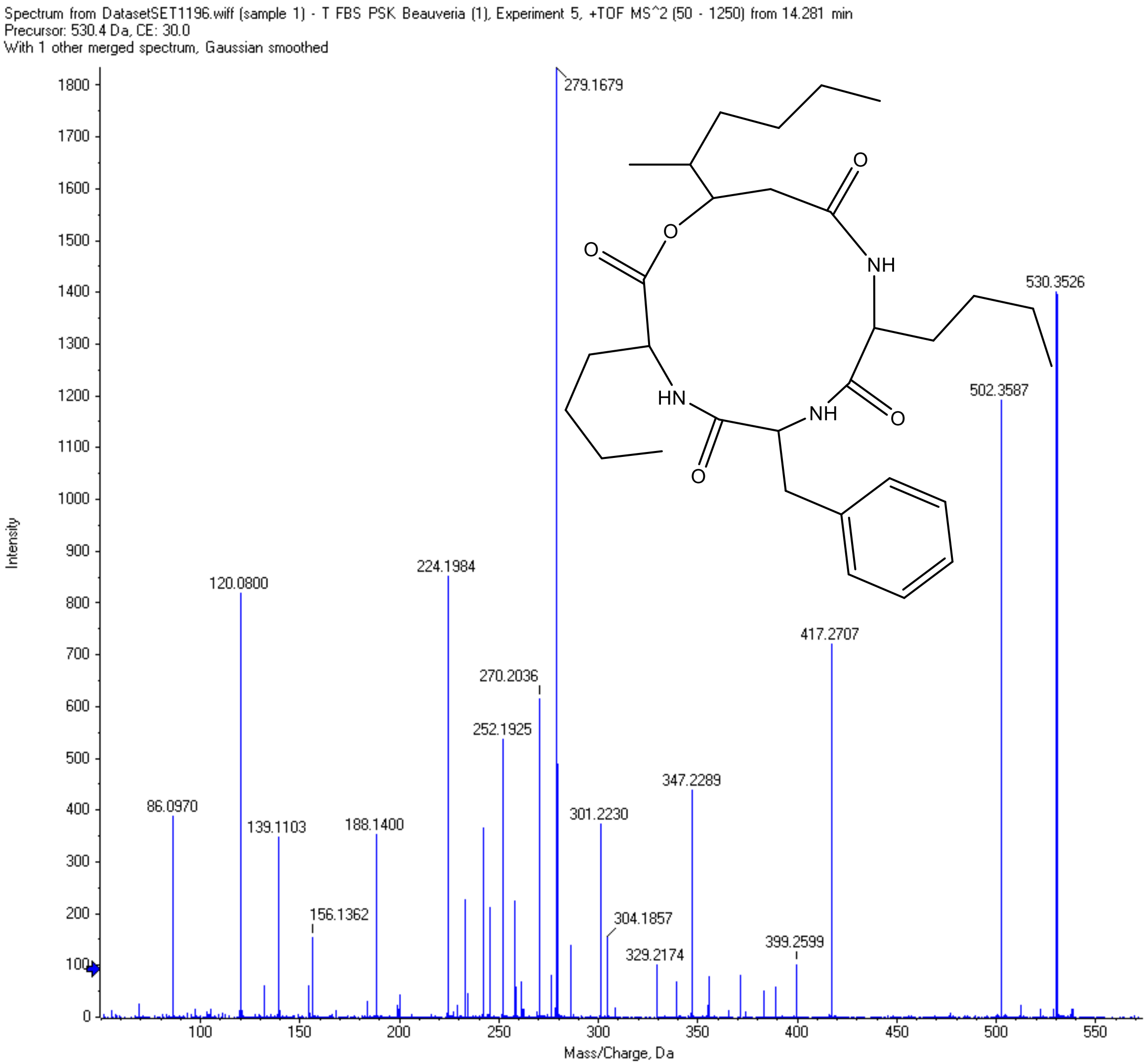
Spectrum from DatasetSET1196.wiff (sample 1) - T FBS PSK Beauveria (1), Experiment 5, +TOF MS^2 (50 - 1250) from 14.281 min
Precursor: 530.4 Da, CE: 30.0
With 1 other merged spectrum, Gaussian smoothed
Intensity
Mass/Charge, Da
279.1679
530.3526
502.3587
417.2707
224.1984
120.0800
270.2036
252.1925
347.2289
86.0970
301.2230
139.1103
188.1400
156.1362
304.1857
329.2174
399.2599
O
NH
HN

Spectrum from DatasetSET1196.wiff (sample 1) - T FBS PSK Beauveria (1), Experiment 5, +TOF MS^2 (50 - 1250) from 13.471 min
Precursor: 532.3 Da, CE: 30.0
With 1 other merged spectrum, Gaussian smoothed

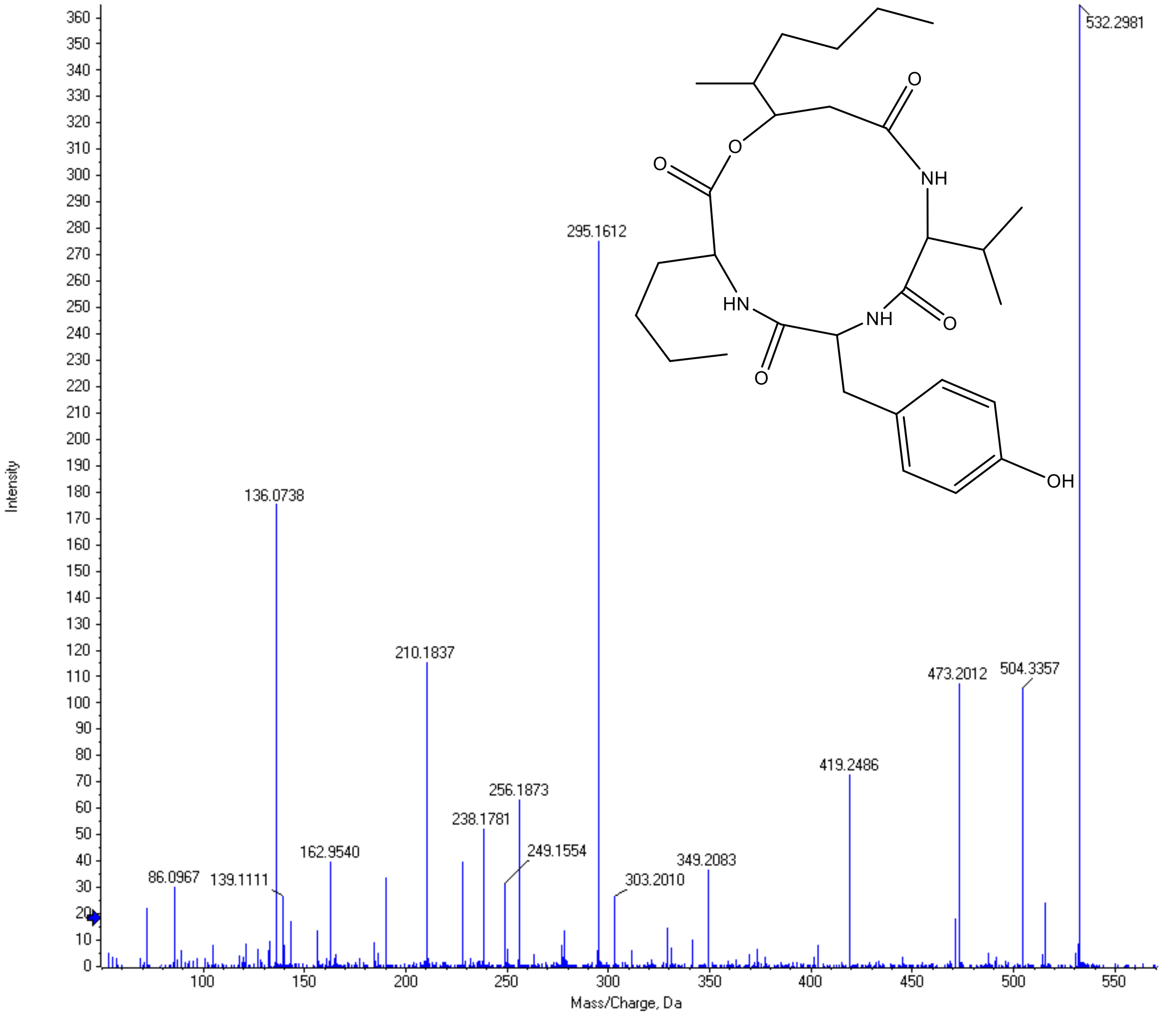

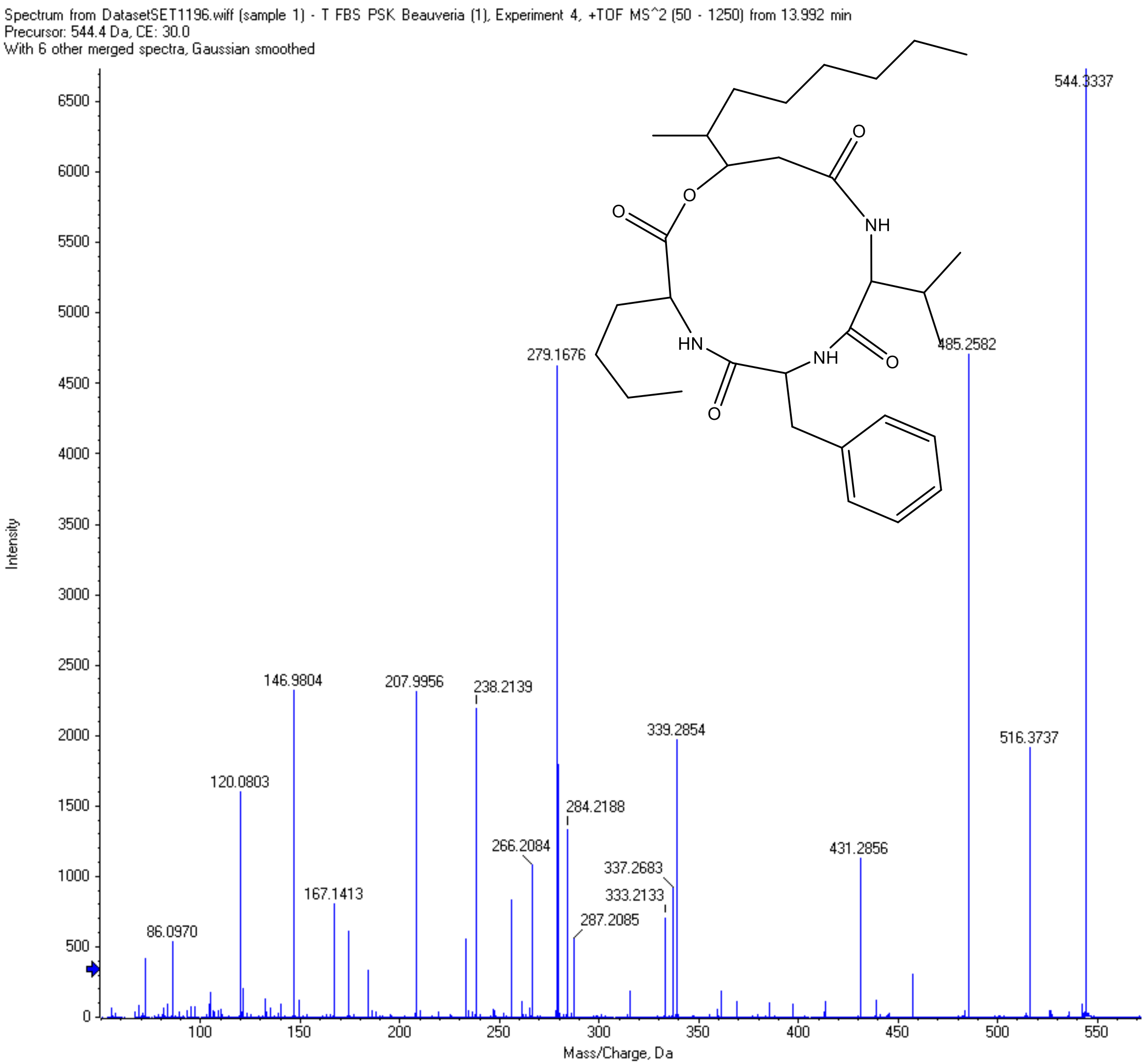

Spectrum from DatasetSET1196.wiff (sample 1) - T FBS PSK Beauveria (1), Experiment 4, +TOF MS^2 (50 - 1250) from 13.992 min
Precursor: 544.4 Da, CE: 30.0
With 6 other merged spectra, Gaussian smoothed
Intensity
Mass/Charge, Da
544.3337
485.2582
279.1676
146.9804
207.9956
238.2139
339.2854
516.3737
120.0803
284.2188
266.2084
431.2856
337.2683
167.1413
333.2133
287.2085
86.0970
O
NH
HN

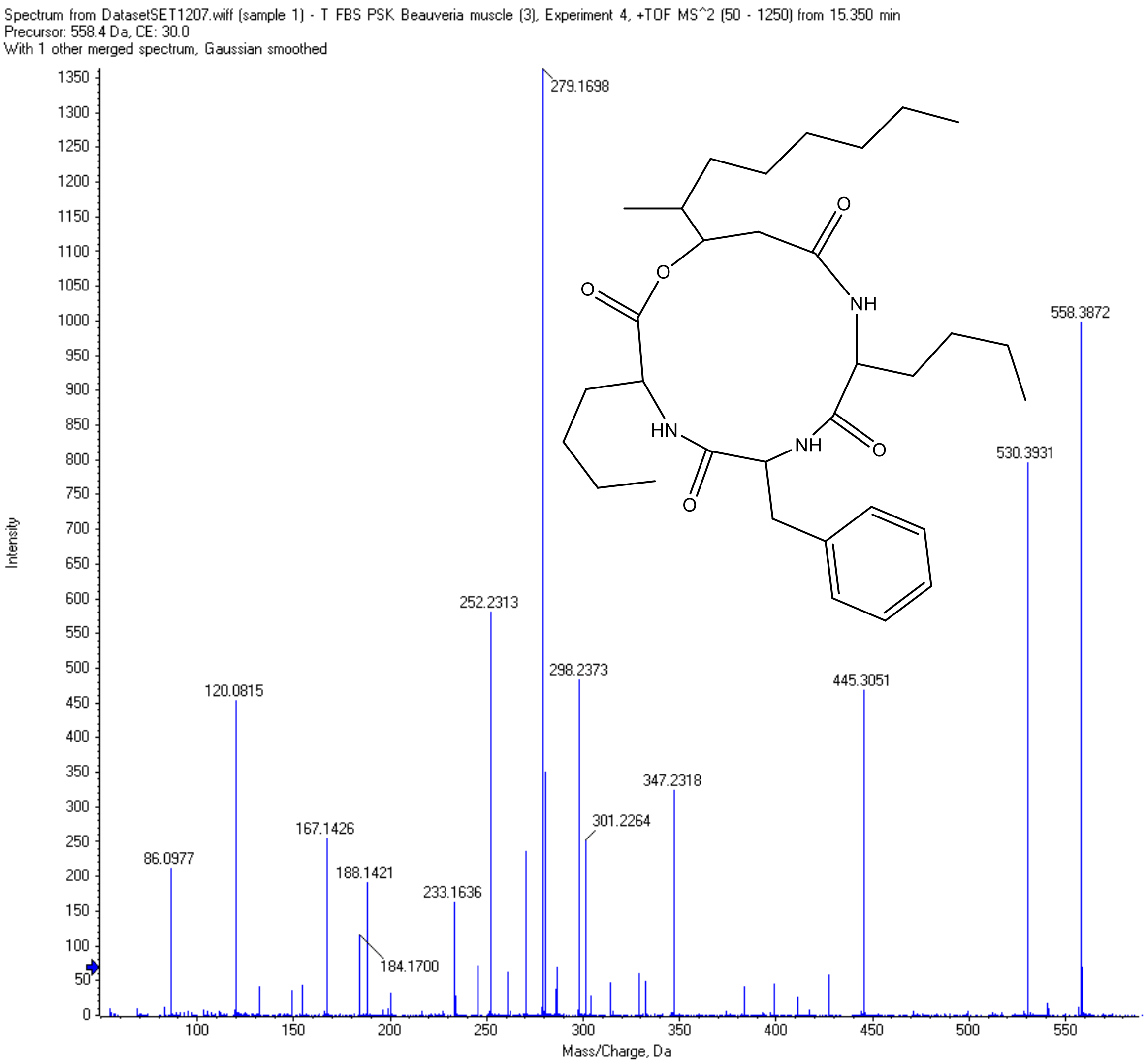
Spectrum from DatasetSET1207.wiff (sample 1) - T FBS PSK Beauveria muscle (3), Experiment 4, +TOF MS^2 (50 - 1250) from 15.350 min
Precursor: 558.4 Da, CE: 30.0
With 1 other merged spectrum, Gaussian smoothed
Intensity
Mass/Charge, Da
279.1698
558.3872
530.3931
252.2313
298.2373
445.3051
120.0815
347.2318
301.2264
167.1426
86.0977
188.1421
233.1636
184.1700
O
NH
HN
NH

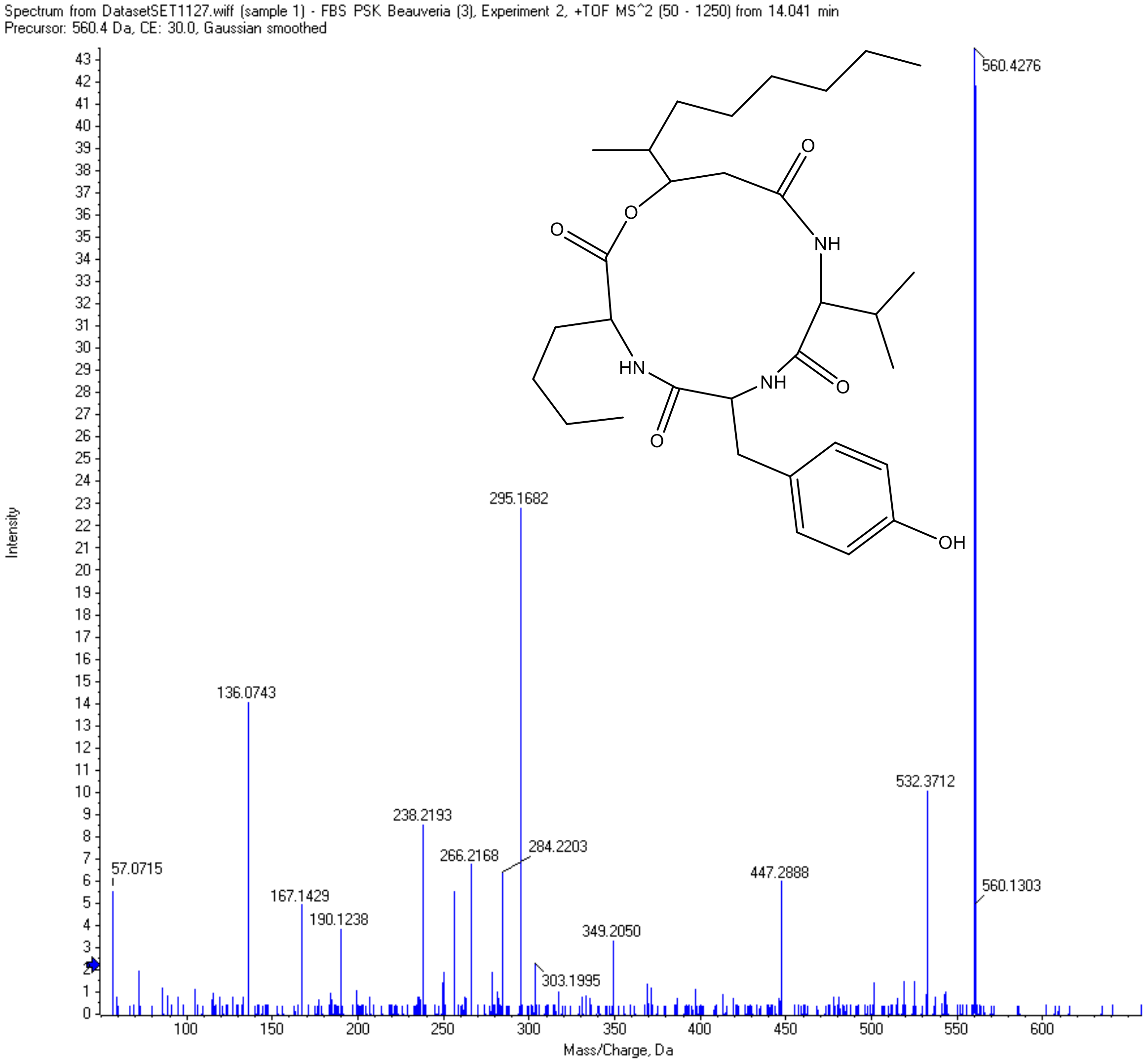

Spectrum from DatasetSET1127.wiff (sample 1) - FBS PSK Beauveria (3), Experiment 2, +TOF MS^2 (50 - 1250) from 14.041 min
Precursor: 560.4 Da, CE: 30.0, Gaussian smoothed
Intensity
Mass/Charge, Da
560.4276
295.1682
136.0743
532.3712
238.2193
266.2168
284.2203
447.2888
560.1303
57.0715
167.1429
190.1238
349.2050
303.1995
O
NH
HN
OH

Spectrum from DatasetSET1196.wiff (sample 1) - T FBS PSK Beauveria (1), Experiment 4, +TOF MS^2 (50 - 1250) from 14.227 min
Precursor: 564.3 Da, CE: 30.0
With 1 other merged spectrum, Gaussian smoothed

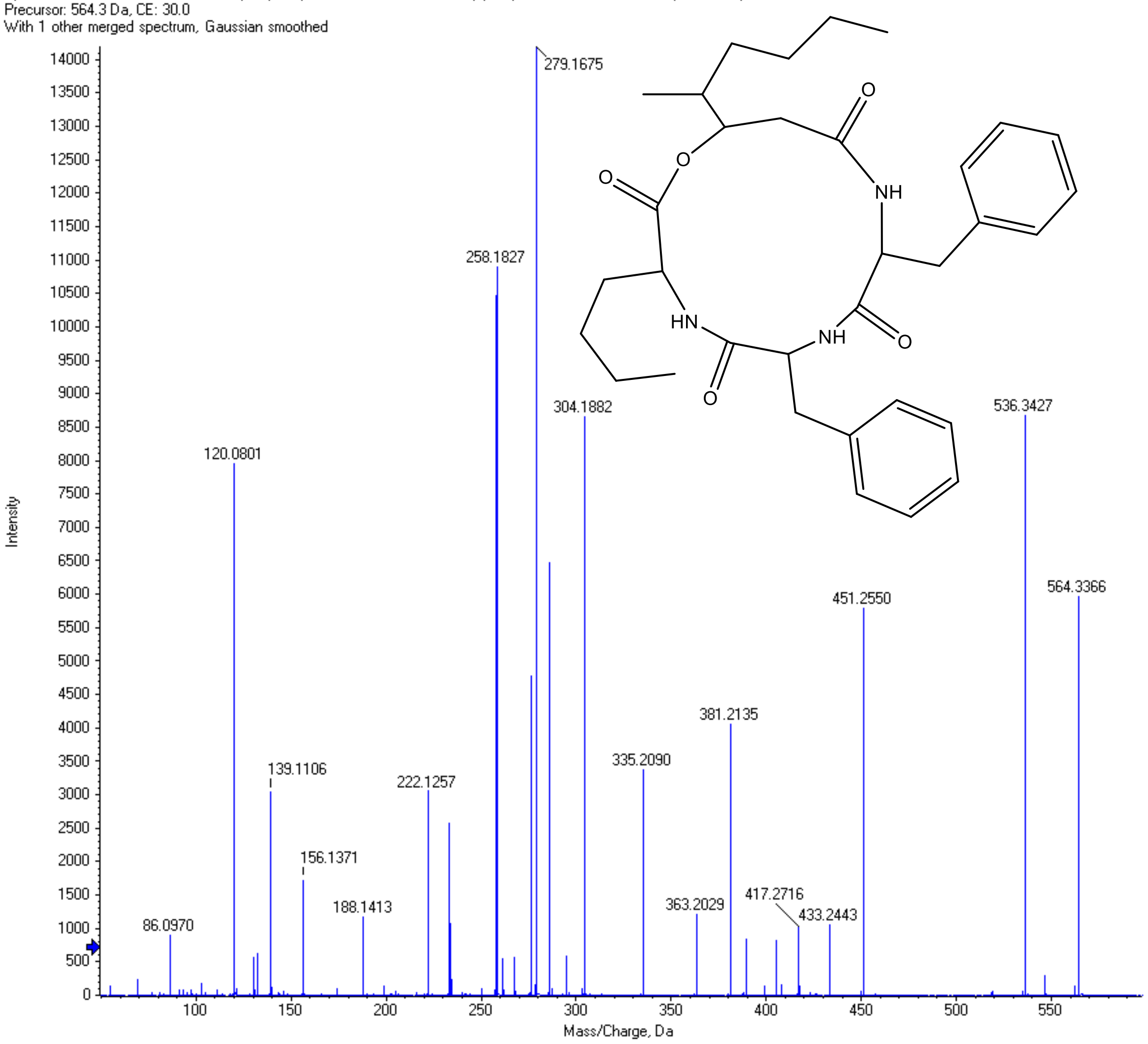

Spectrum from DatasetSET1196.wiff (sample 1) - T FBS PSK Beauveria (1), Experiment 3, +TOF MS^2 (50 - 1250) from 14.697 min
Precursor: 576.2 Da, CE: 30.0, Gaussian smoothed

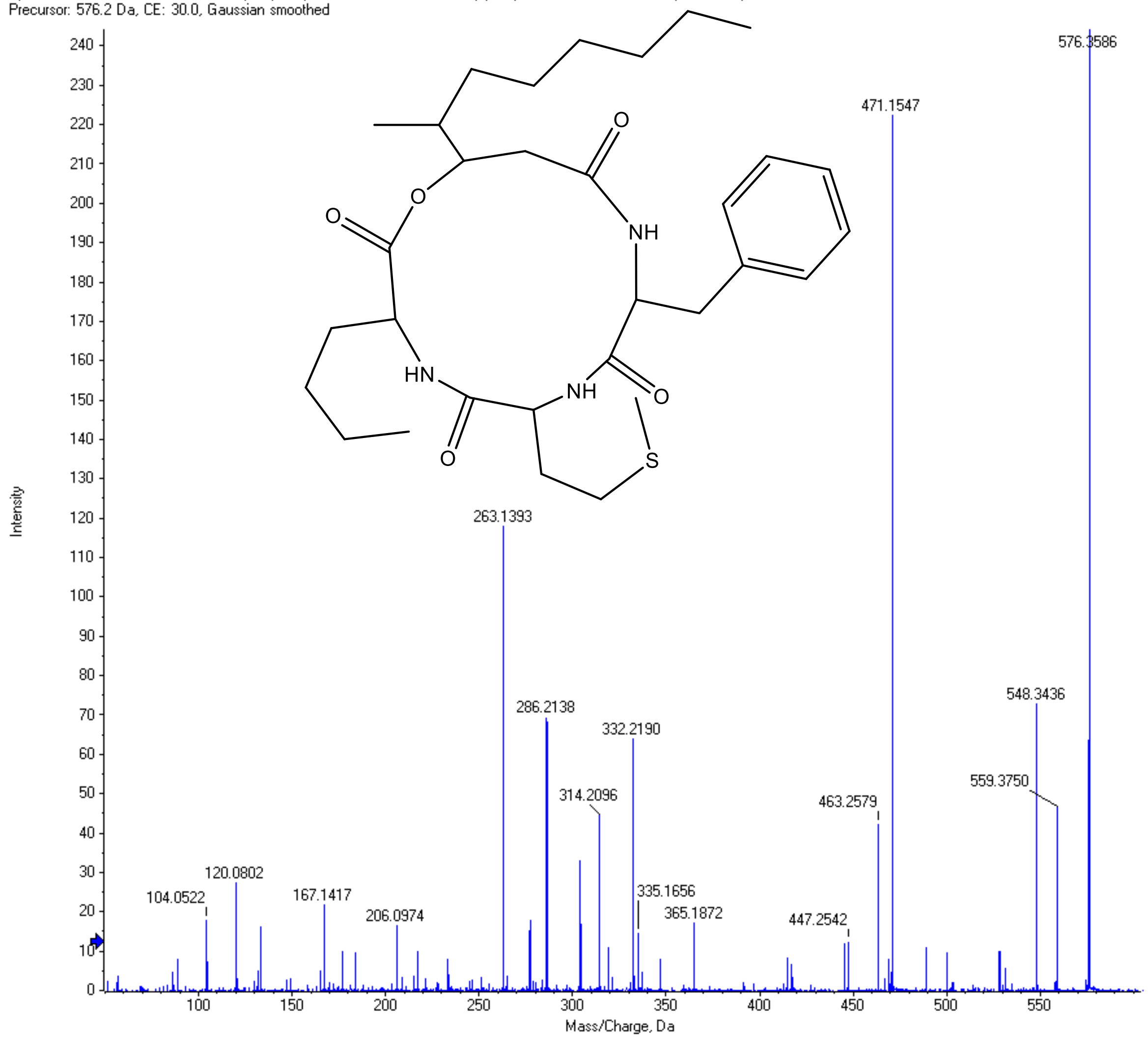

Spectrum from DatasetSET1150.wiff (sample 1) - FBS PSK Beauveria muscle (2), Experiment 4, +TOF MS^2 (50 - 1250) from 13.538 min
Precursor: 580.3 Da, CE: 30.0, Gaussian smoothed

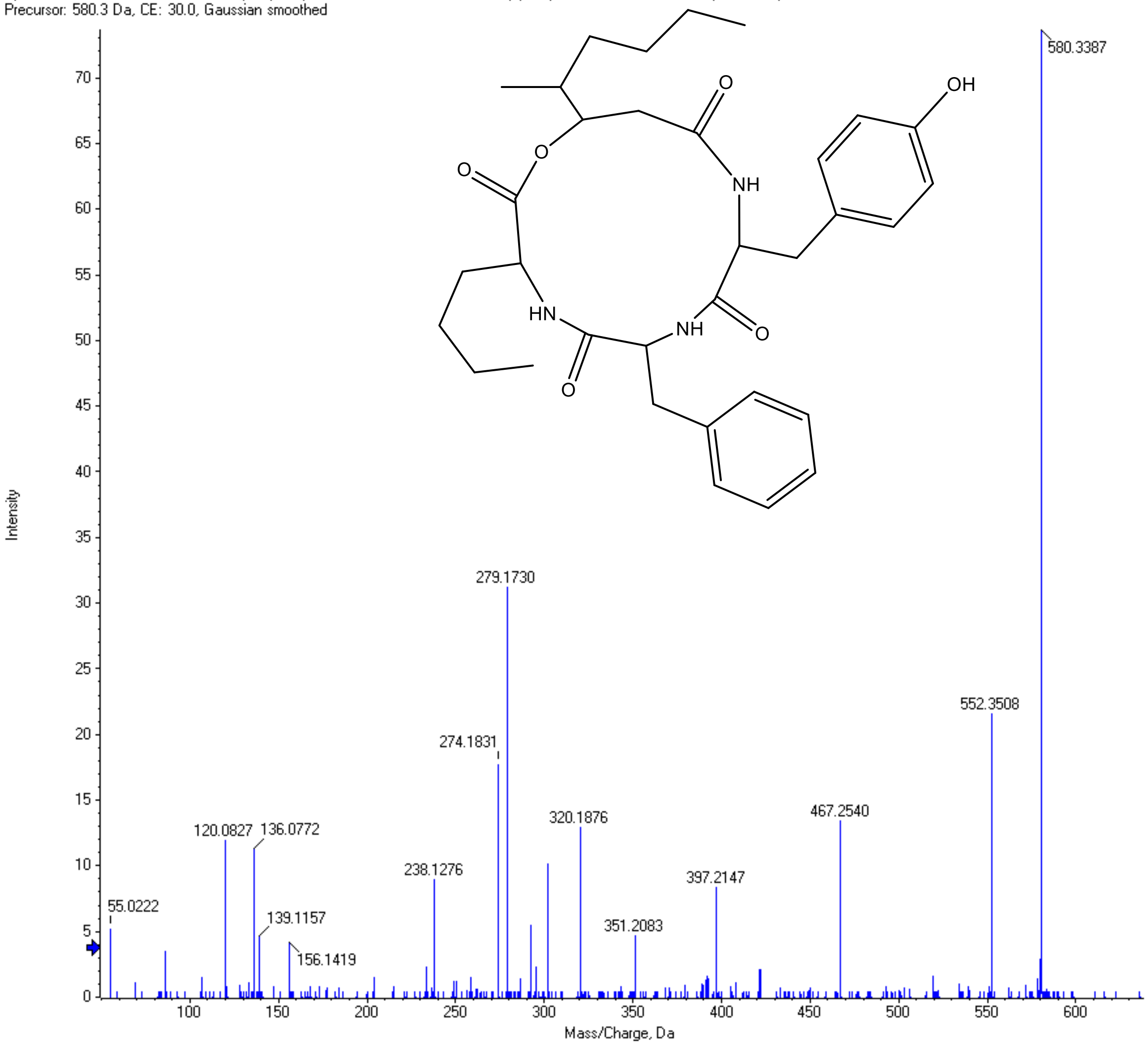

Spectrum from DatasetSET1207.wiff (sample 1) - T FBS PSK Beauveria muscle (3), Experiment 3, +TOF MS^2 (50 - 1250) from 15.322 min
Precursor: 592.4 Da, CE: 30.0
With 1 other merged spectrum, Gaussian smoothed

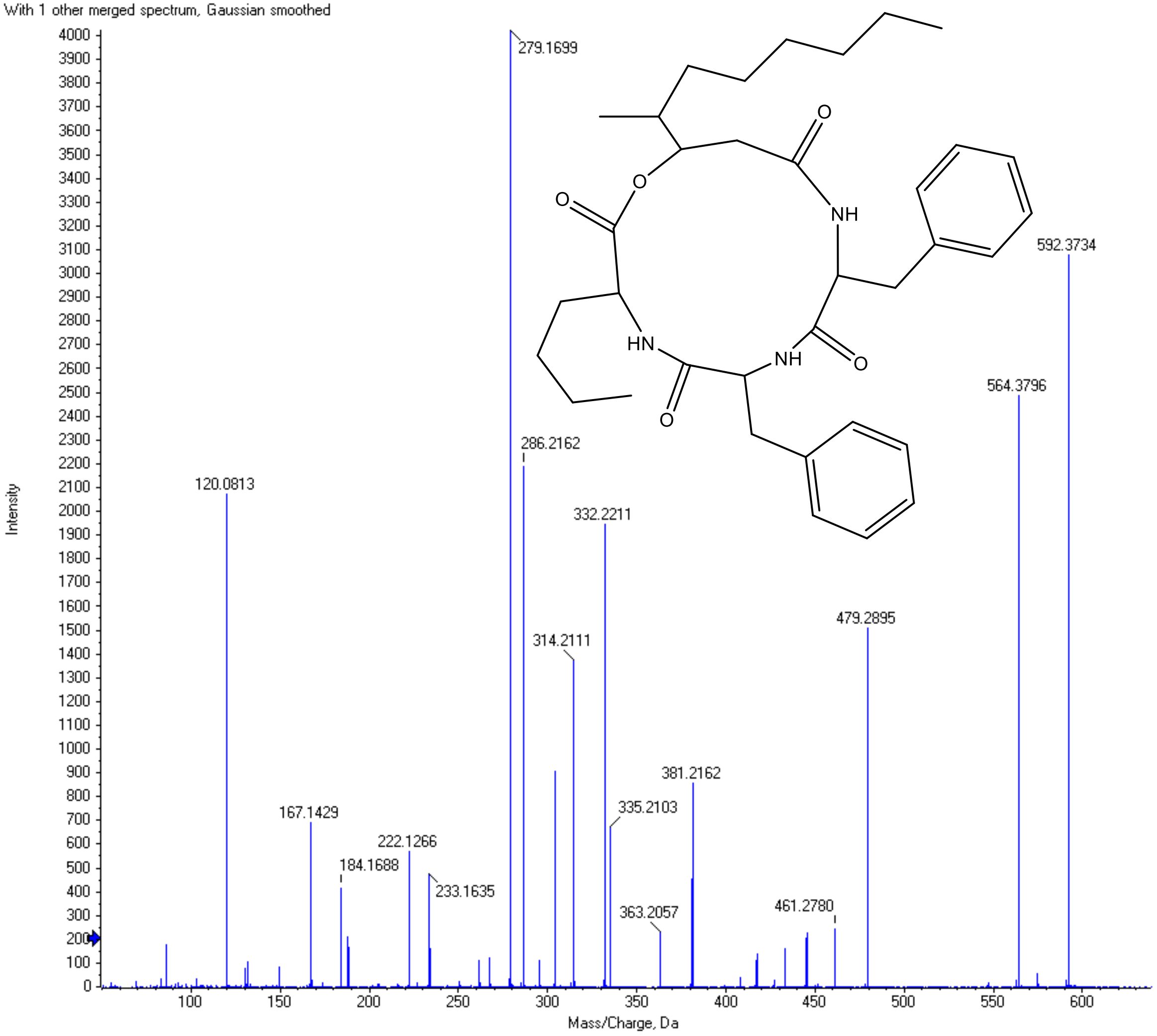

Spectrum from DatasetSET1150.wiff (sample 1) - FBS PSK Beauveria muscle (2), Experiment 2, +TOF MS^2 (50 - 1250) from 14.080 min
Precursor: 603.4 Da, CE: 30.0
With 1 other merged spectrum, Gaussian smoothed

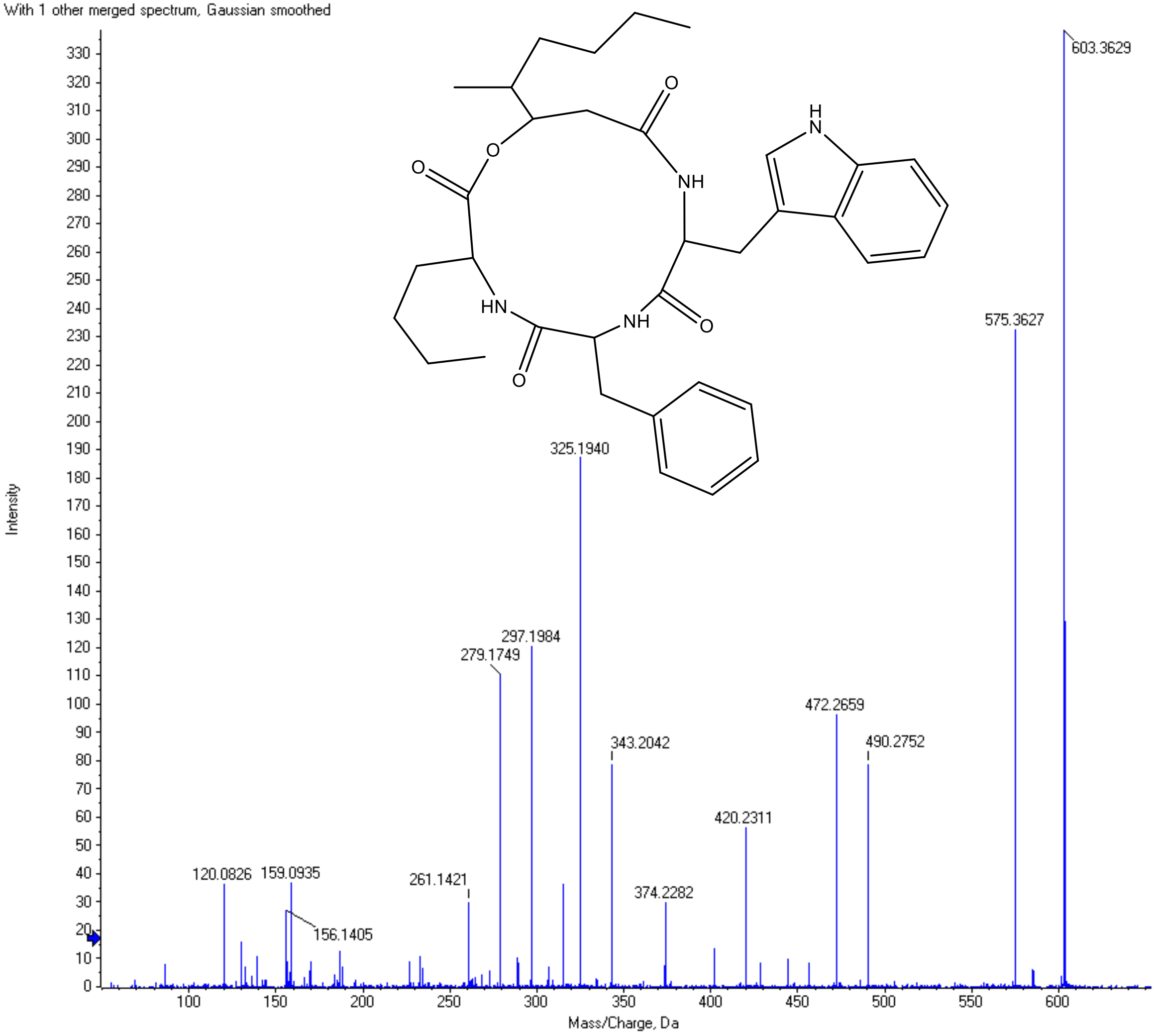

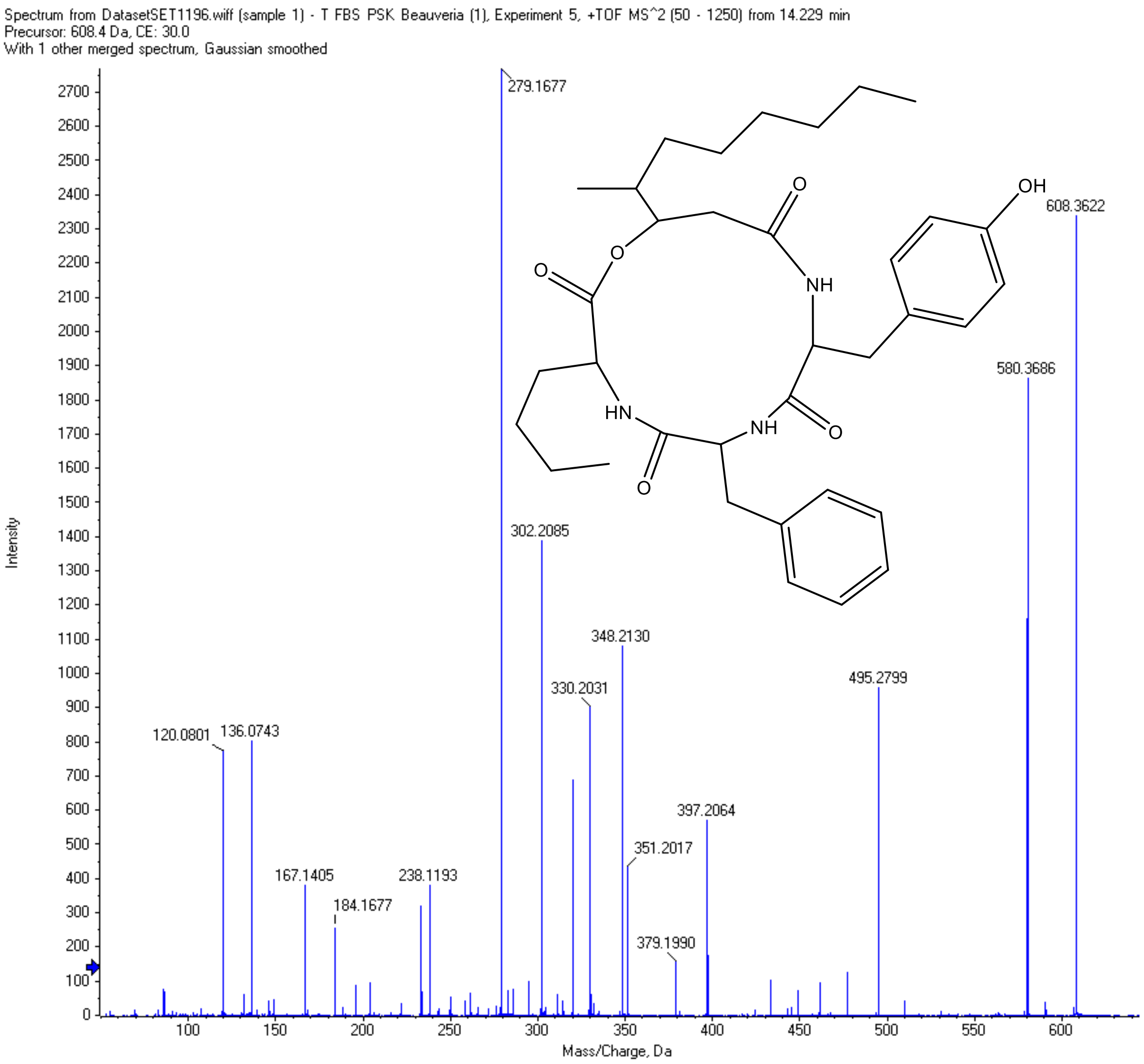

Spectrum from DatasetSET1196.wiff (sample 1) - T FBS PSK Beauveria (1), Experiment 5, +TOF MS^2 (50 - 1250) from 14.229 min
Precursor: 608.4 Da, CE: 30.0
With 1 other merged spectrum, Gaussian smoothed
Intensity
Mass/Charge, Da
279.1677
608.3622
580.3686
302.2085
348.2130
330.2031
495.2799
120.0801
136.0743
397.2064
351.2017
167.1405
238.1193
184.1677
379.1990
OH
O
O
O
NH
HN
NH
O
O

Spectrum from DatasetSET1207.wiff (sample 1) - T FBS PSK Beauveria muscle (3), Experiment 5, +TOF MS^2 (50 - 1250) from 14.835 min
Precursor: 615.4 Da, CE: 30.0
With 2 other merged spectra, Gaussian smoothed

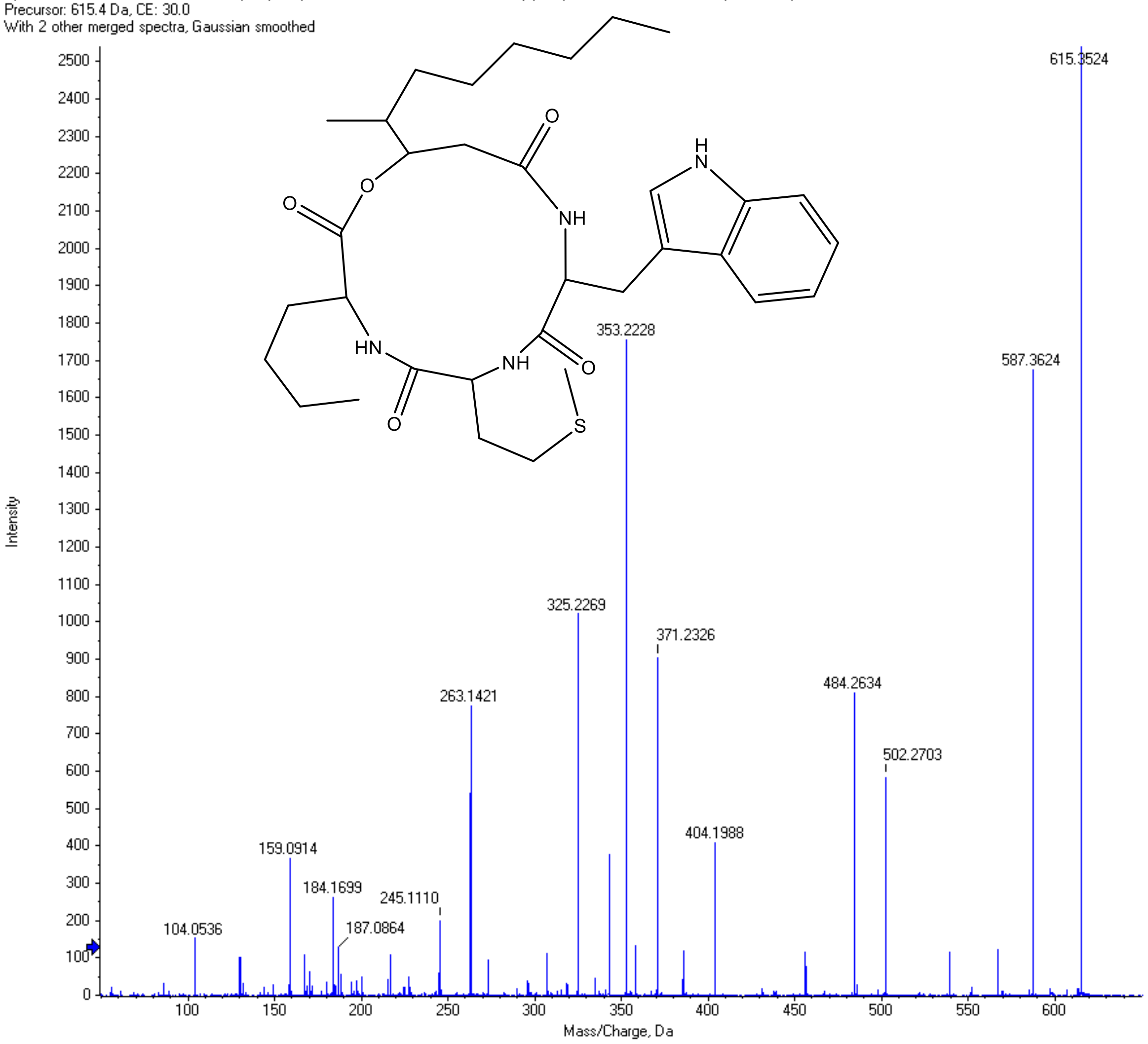

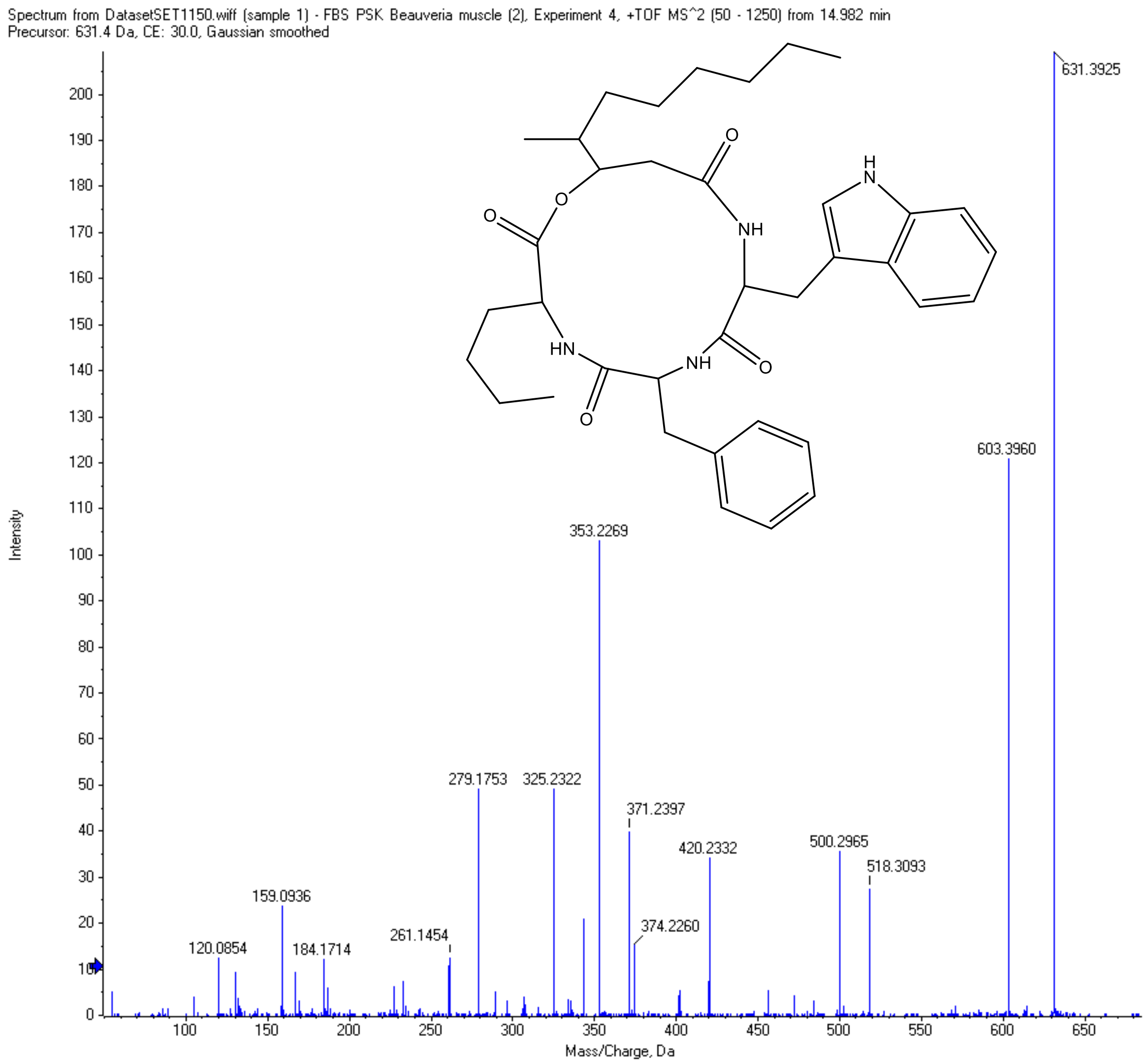

Spectrum from DatasetSET1150.wiff (sample 1) - FBS PSK Beauveria muscle (2), Experiment 4, +TOF MS^2 (50 - 1250) from 14.982 min
Precursor: 631.4 Da, CE: 30.0, Gaussian smoothed
Intensity
Mass/Charge, Da
631.3925
603.3960
353.2269
279.1753
325.2322
371.2397
420.2332
500.2965
518.3093
374.2260
159.0936
120.0854
184.1714
261.1454
O
NH
HN
H
N

Destruxins

Product ion mass spectra of the 14 destruxins found in this study in the order as described in Table 2

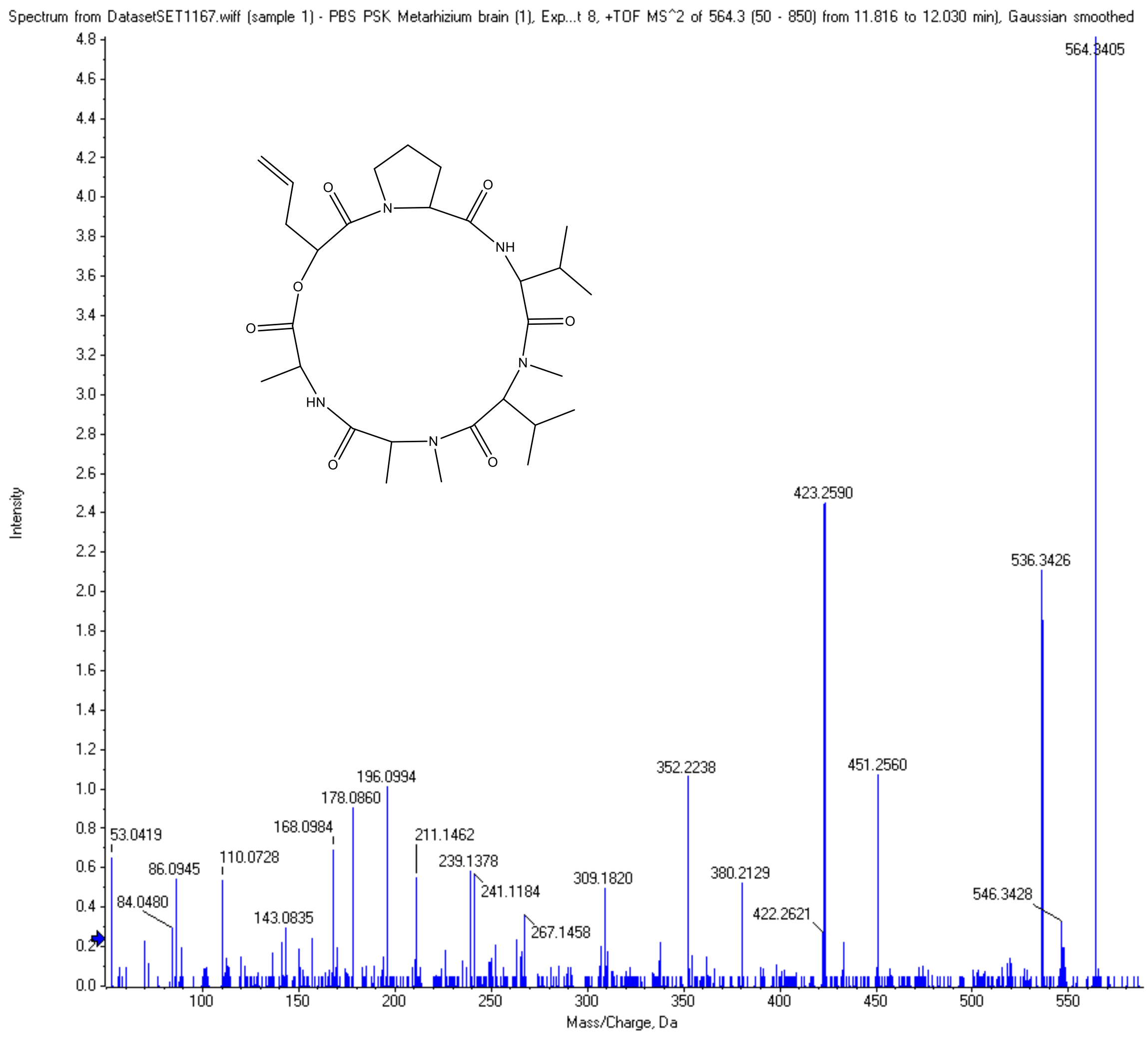

Spectrum from DatasetSET1167.wiff (sample 1) - PBS PSK Metarhizium brain (1), Exp...t 8, +TOF MS^2 of 564.3 (50 - 850) from 11.816 to 12.030 min), Gaussian smoothed
Intensity
Mass/Charge, Da
564.3405
423.2590
536.3426
352.2238
451.2560
196.0994
178.0860
168.0984
211.1462
53.0419
239.1378
110.0728
86.0945
241.1184
309.1820
380.2129
84.0480
546.3428
422.2621
143.0835
267.1458

Spectrum from DatasetSET1167.wiff (sample 1) - PBS PSK Metarhizium brain (1), Ex...nt 8, +TOF MS^2 of 564.3 (50 - 850) from 10.853 to 10.943 min, Gaussian smoothed

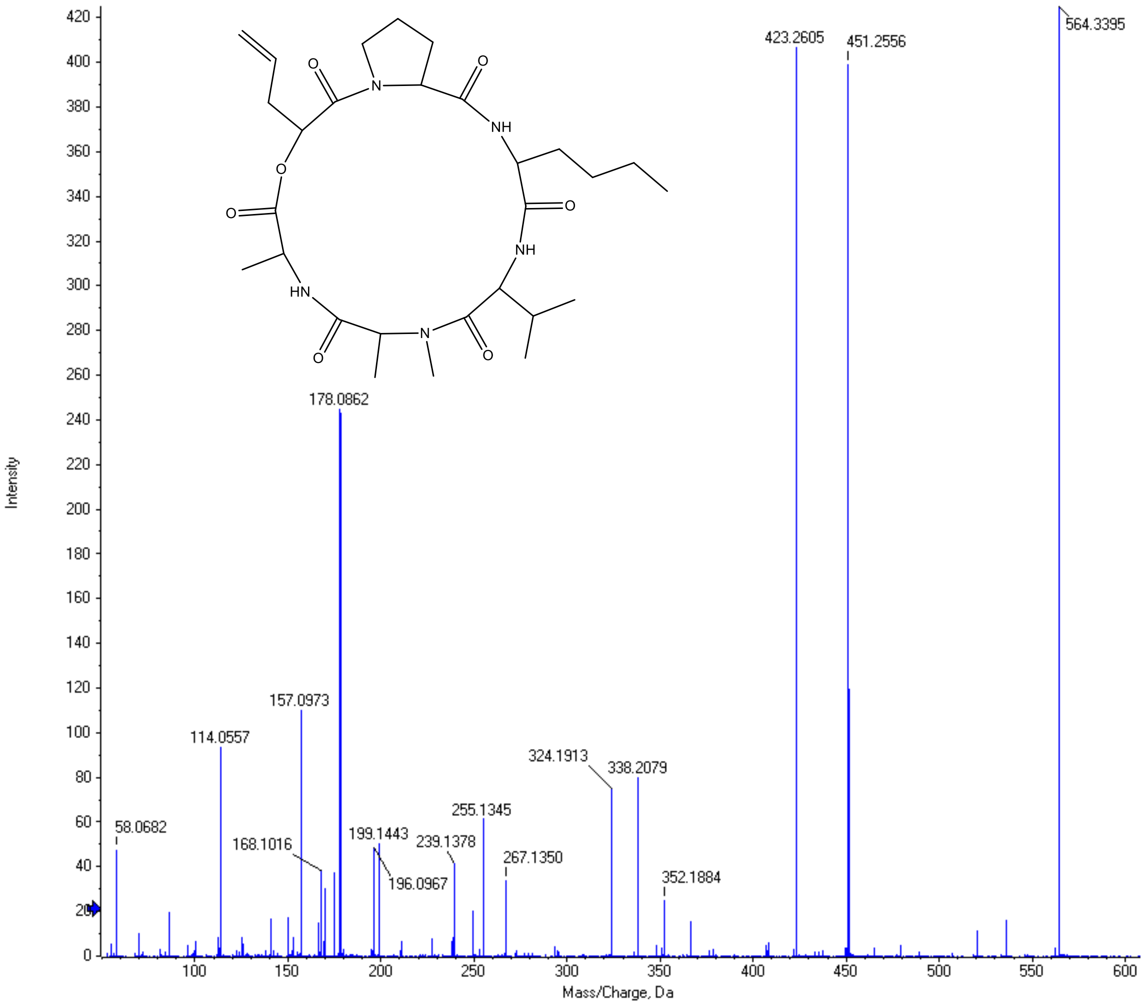

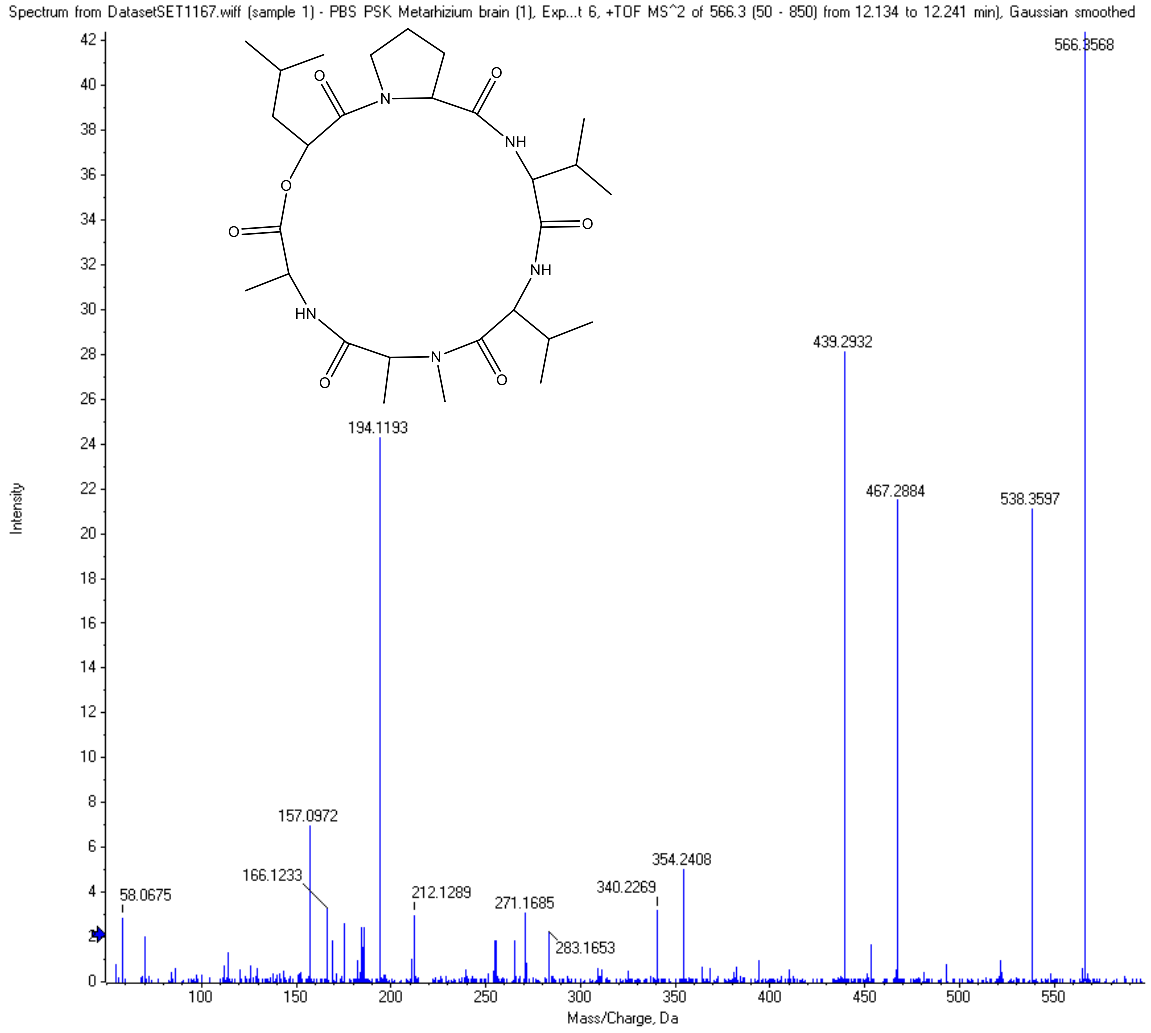

Spectrum from DatasetSET1167.wiff (sample 1) - PBS PSK Metarhizium brain (1), Exp...t 6, +TOF MS^2 of 566.3 (50 - 850) from 12.134 to 12.241 min), Gaussian smoothed
Intensity
Mass/Charge, Da
566.3568
439.2932
467.2884
538.3597
194.1193
157.0972
166.1233
58.0675
212.1289
271.1685
283.1653
340.2269
354.2408

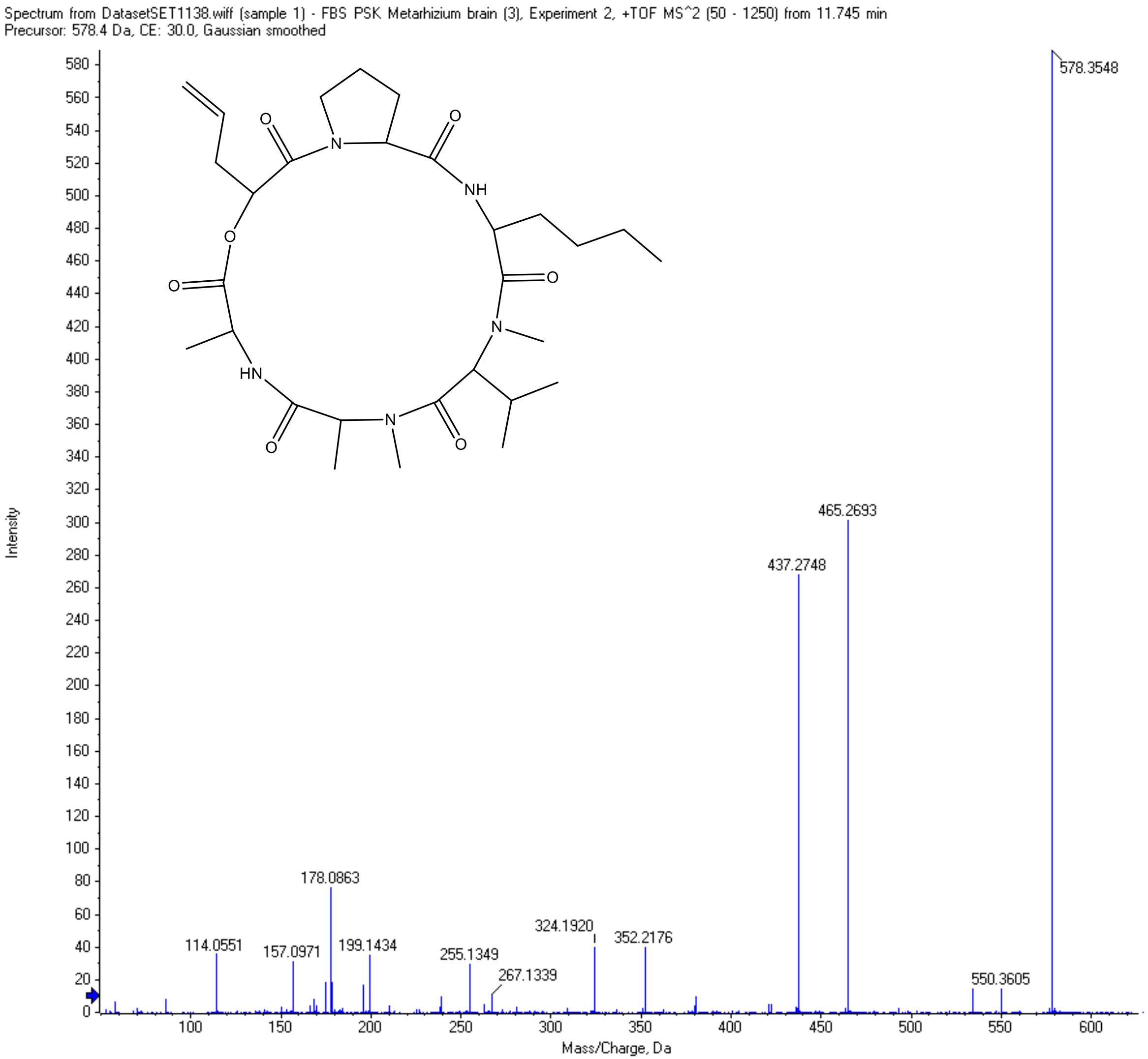
Spectrum from DatasetSET1138.wiff (sample 1) - FBS PSK Metarhizium brain (3), Experiment 2, +TOF MS^2 (50 - 1250) from 11.745 min
Precursor: 578.4 Da, CE: 30.0, Gaussian smoothed
578.3548
465.2693
437.2748
178.0863
114.0551
157.0971
199.1434
255.1349
267.1339
324.1920
352.2176
550.3605
Intensity
Mass/Charge, Da

Spectrum from DatasetSET1109.wiff (sample 1) - PBS PSK Metarhizium brain (1), Experiment 3, +TOF MS^2 (50 - 1250) from 9.312 min
Precursor: 580.3 Da, CE: 30.0
With 1 other merged spectrum, Gaussian smoothed

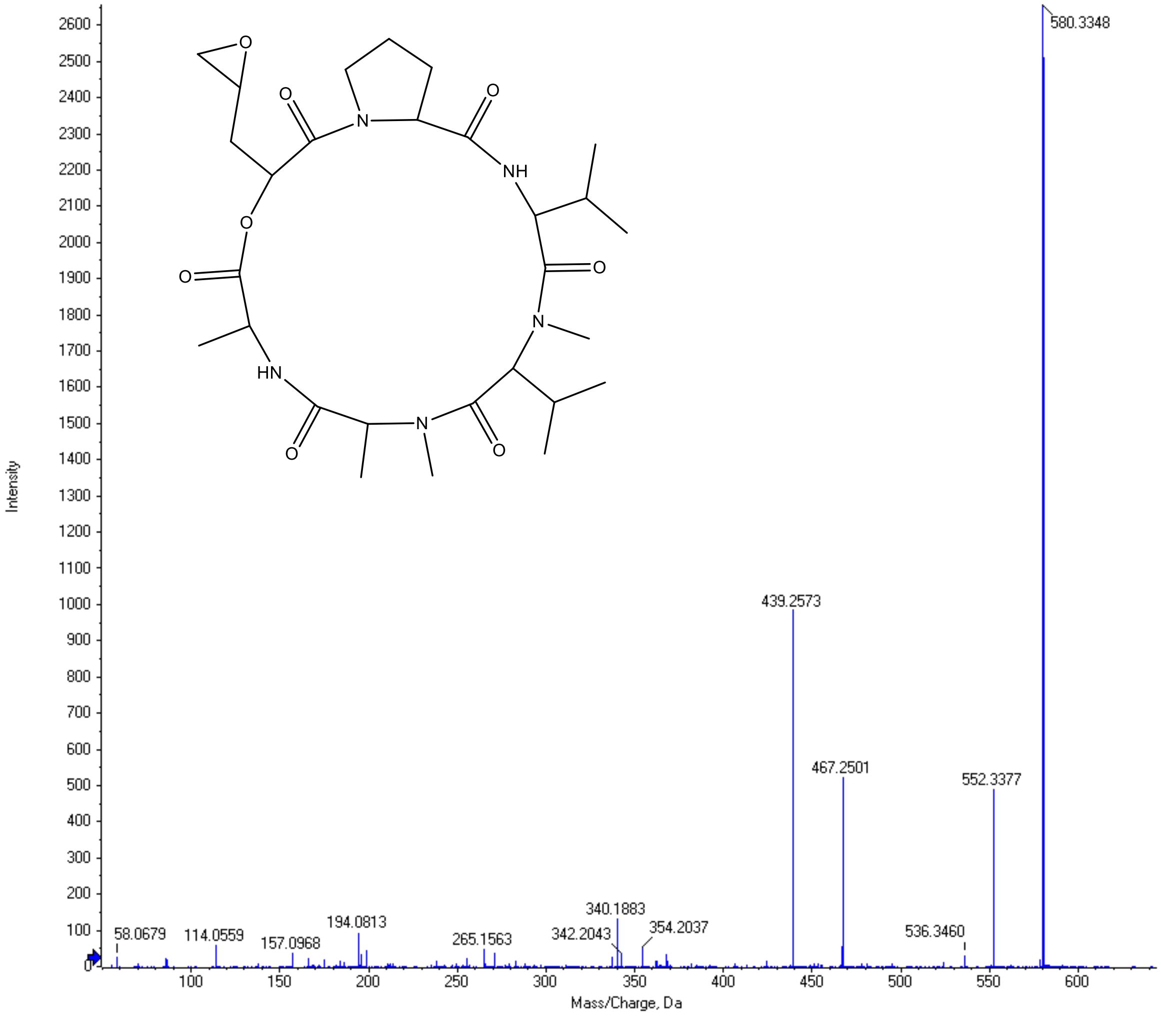

Spectrum from DatasetSET1109.wiff (sample 1) - PBS PSK Metarhizium brain (1), Experiment 2, +TOF MS^2 (50 - 1250) from 12.189 min
Precursor: 580.4 Da, CE: 30.0
With 1 other merged spectrum, Gaussian smoothed

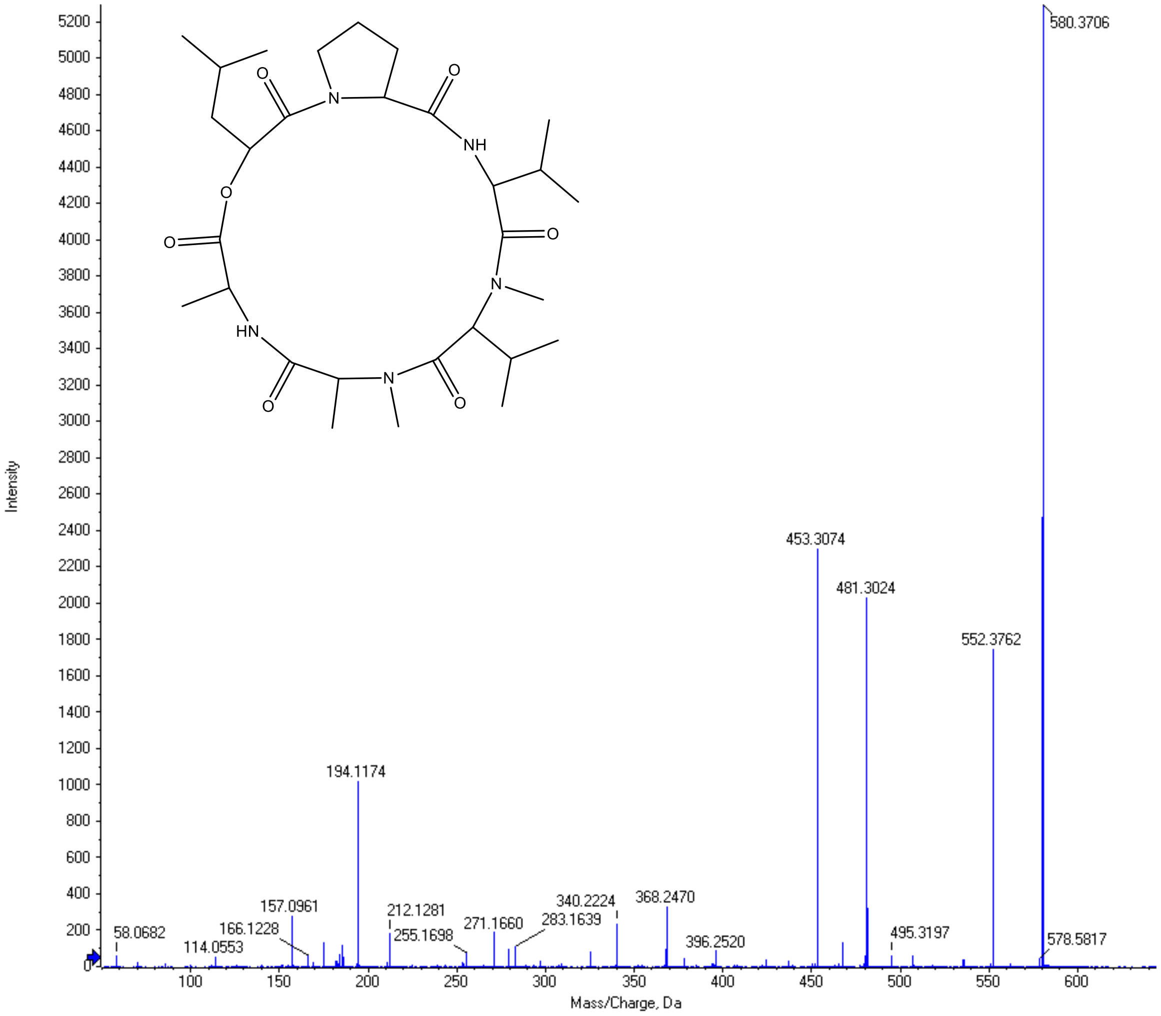

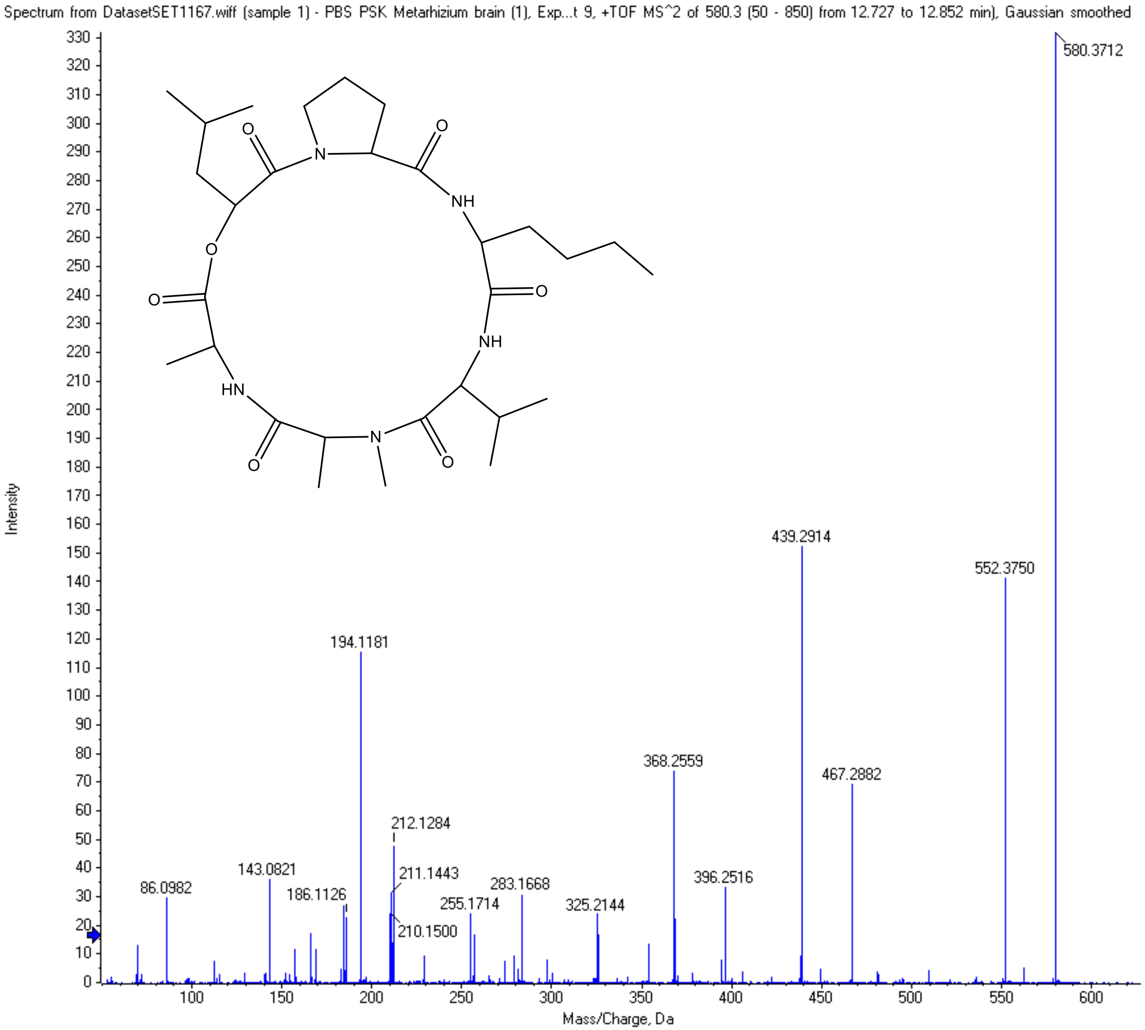

Spectrum from DatasetSET1167.wiff (sample 1) - PBS PSK Metarhizium brain (1), Exp...t 9, +TOF MS^2 of 580.3 (50 - 850) from 12.727 to 12.852 min), Gaussian smoothed
Intensity
Mass/Charge, Da
580.3712
552.3750
439.2914
467.2882
368.2559
396.2516
325.2144
283.1668
255.1714
212.1284
211.1443
210.1500
194.1181
186.1126
143.0821
86.0982

Spectrum from DatasetSET1109.wiff (sample 1) - PBS PSK Metarhizium brain (1), Experiment 3, +TOF MS^2 (50 - 1250) from 12.668 min
Precursor: 594.4 Da, CE: 30.0
With 1 other merged spectrum, Gaussian smoothed

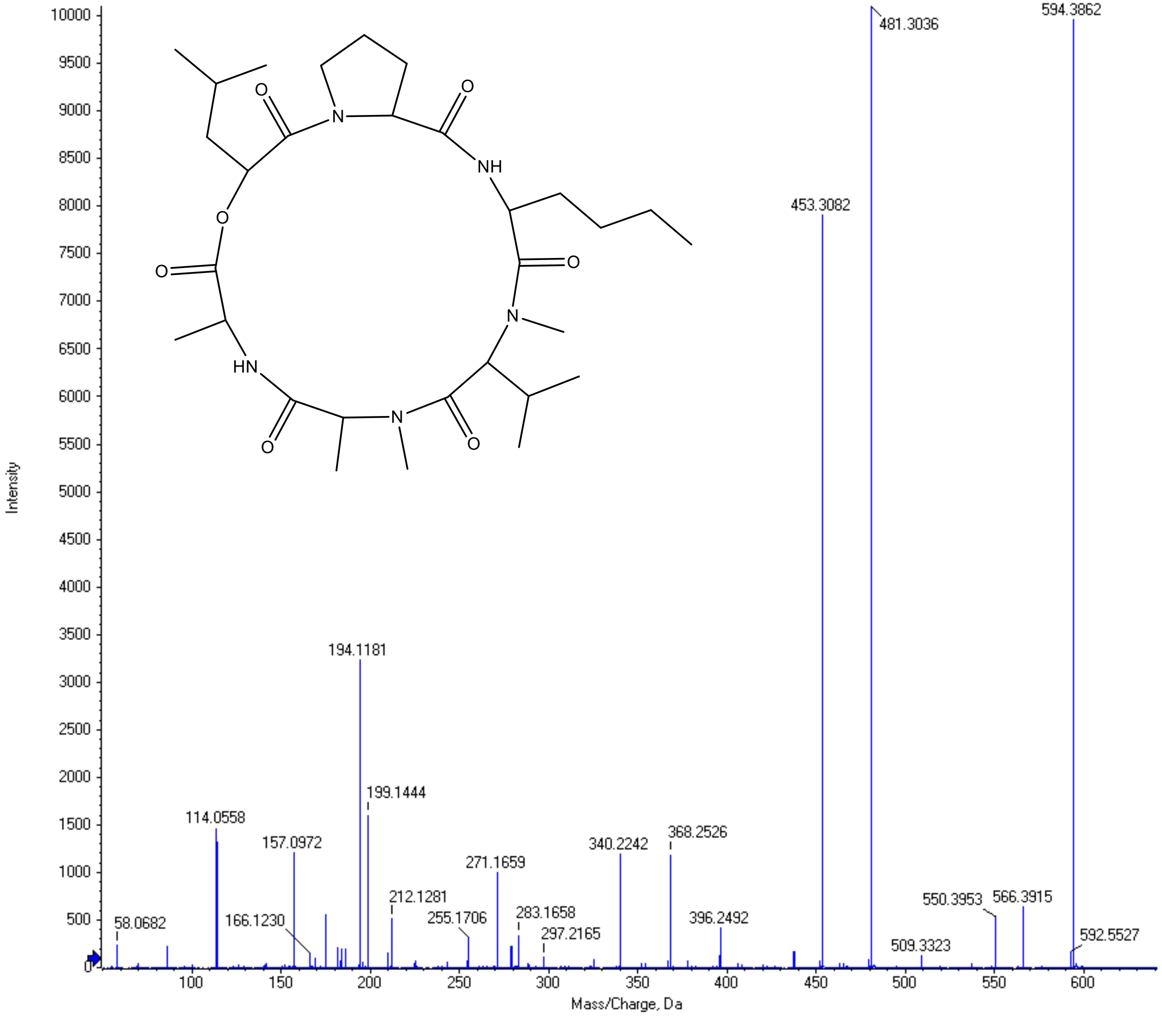

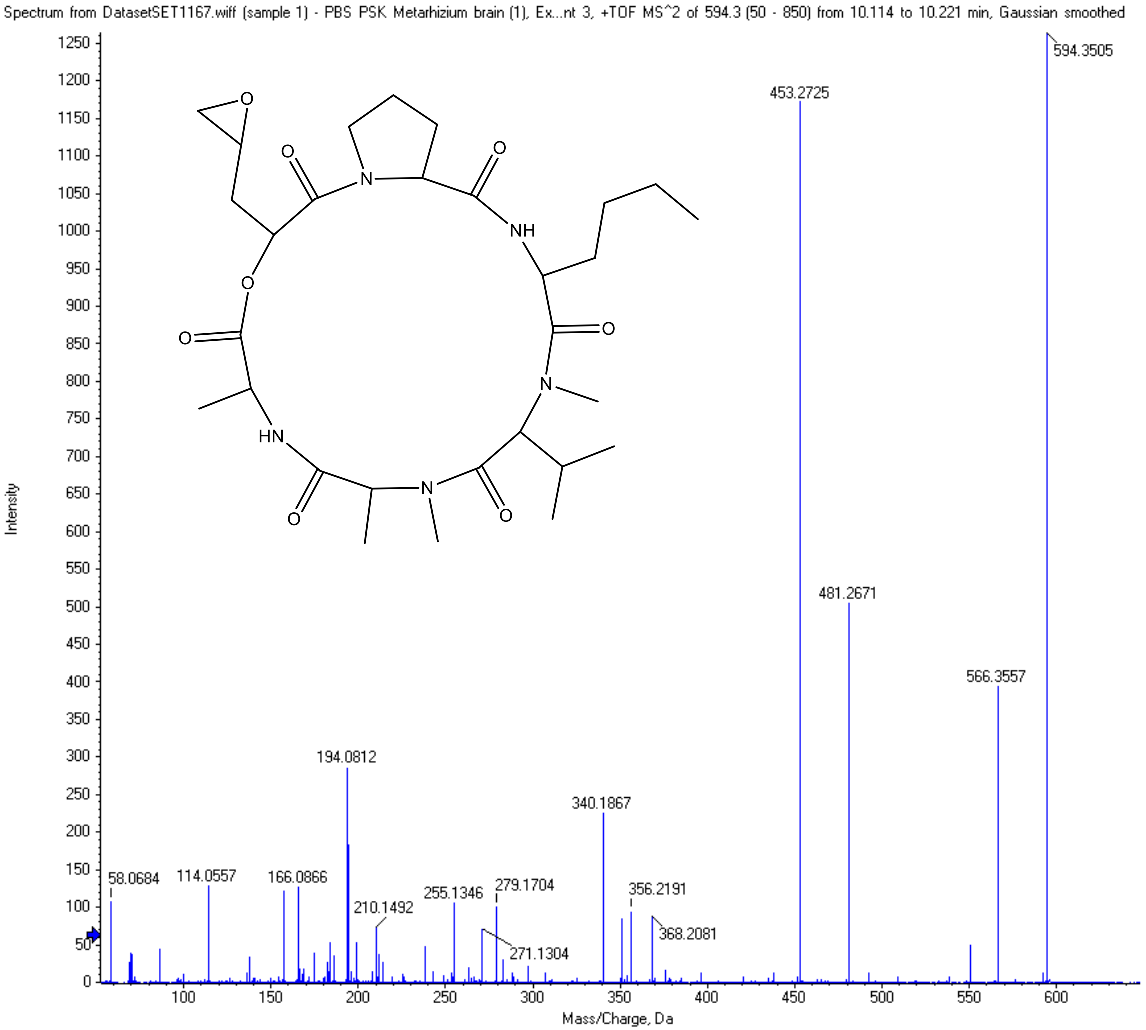

Spectrum from DatasetSET1167.wiff (sample 1) - PBS PSK Metarhizium brain (1), Ex...nt 3, +TOF MS^2 of 594.3 (50 - 850) from 10.114 to 10.221 min, Gaussian smoothed
Intensity
Mass/Charge, Da
594.3505
453.2725
481.2671
566.3557
194.0812
340.1867
58.0684
114.0557
166.0866
210.1492
255.1346
279.1704
356.2191
368.2081
271.1304
O
N
NH
HN

Spectrum from DatasetSET1109.wiff (sample 1) - PBS PSK Metarhizium brain (1), Experiment 2, +TOF MS^2 (50 - 1250) from 9.632 min
Precursor: 596.4 Da, CE: 30.0
With 2 other merged spectra, Gaussian smoothed

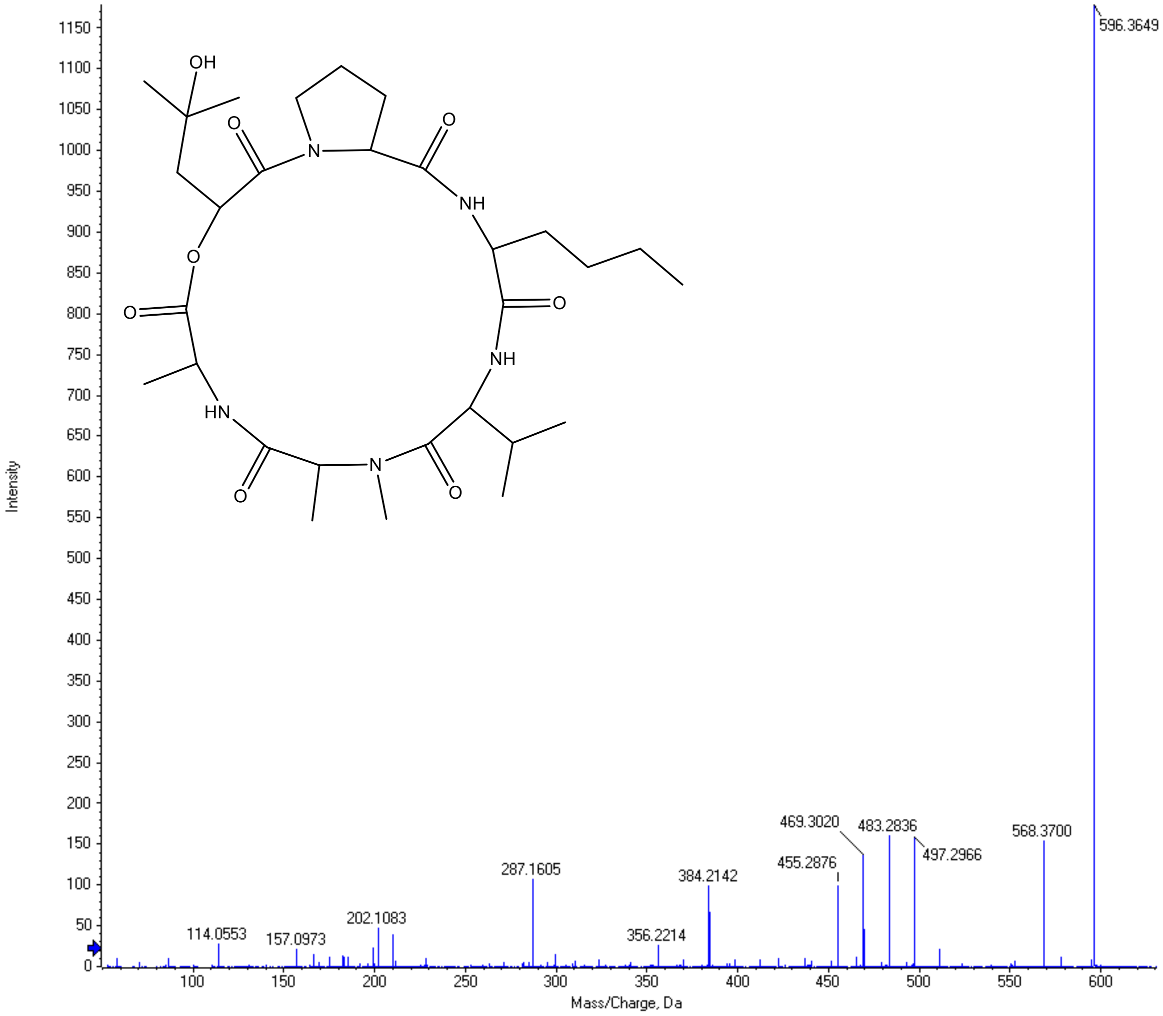

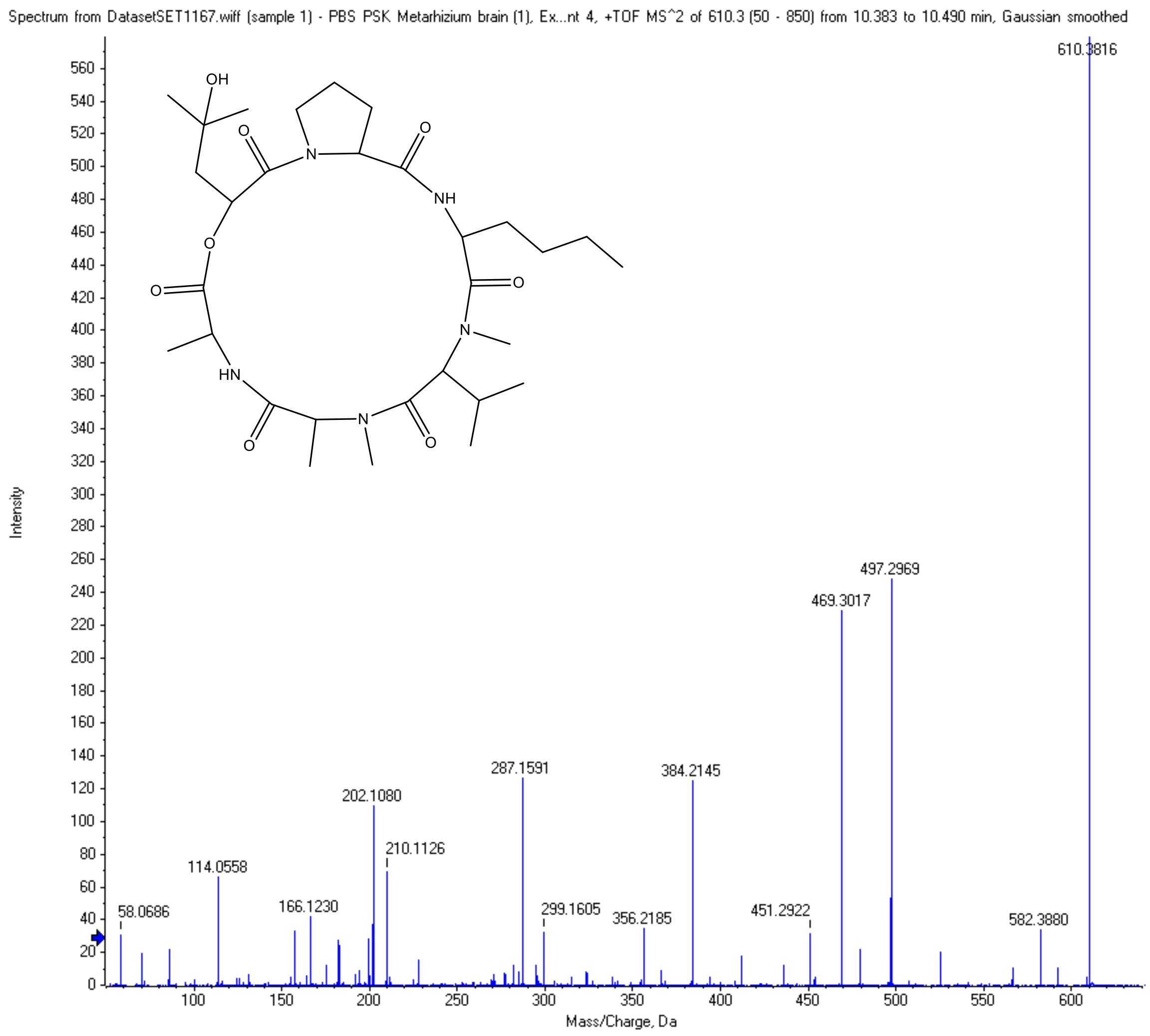

Spectrum from DatasetSET1167.wiff (sample 1) - PBS PSK Metarhizium brain (1), Ex...nt 4, +TOF MS^2 of 610.3 (50 - 850) from 10.383 to 10.490 min, Gaussian smoothed
610.3816
497.2969
469.3017
287.1591
384.2145
202.1080
210.1126
114.0558
166.1230
58.0686
299.1605
356.2185
451.2922
582.3880
Intensity
Mass/Charge, Da

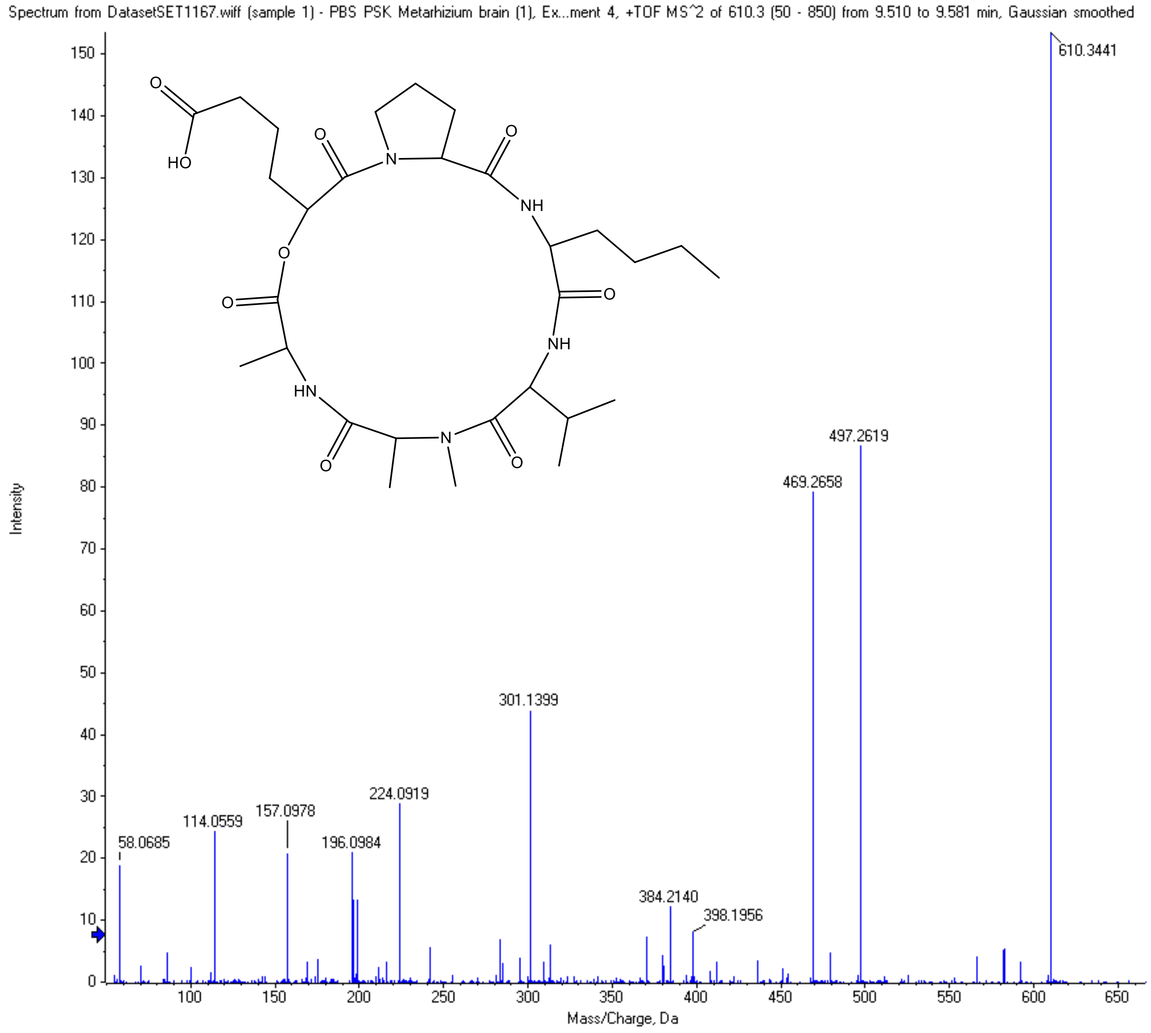
Spectrum from DatasetSET1167.wiff (sample 1) - PBS PSK Metarhizium brain (1), Ex...ment 4, +TOF MS^2 of 610.3 (50 - 850) from 9.510 to 9.581 min, Gaussian smoothed
Intensity
Mass/Charge, Da
610.3441
497.2619
469.2658
301.1399
224.0919
157.0978
114.0559
196.0984
58.0685
384.2140
398.1956
O
HO
N
NH
NH
HN
N
O

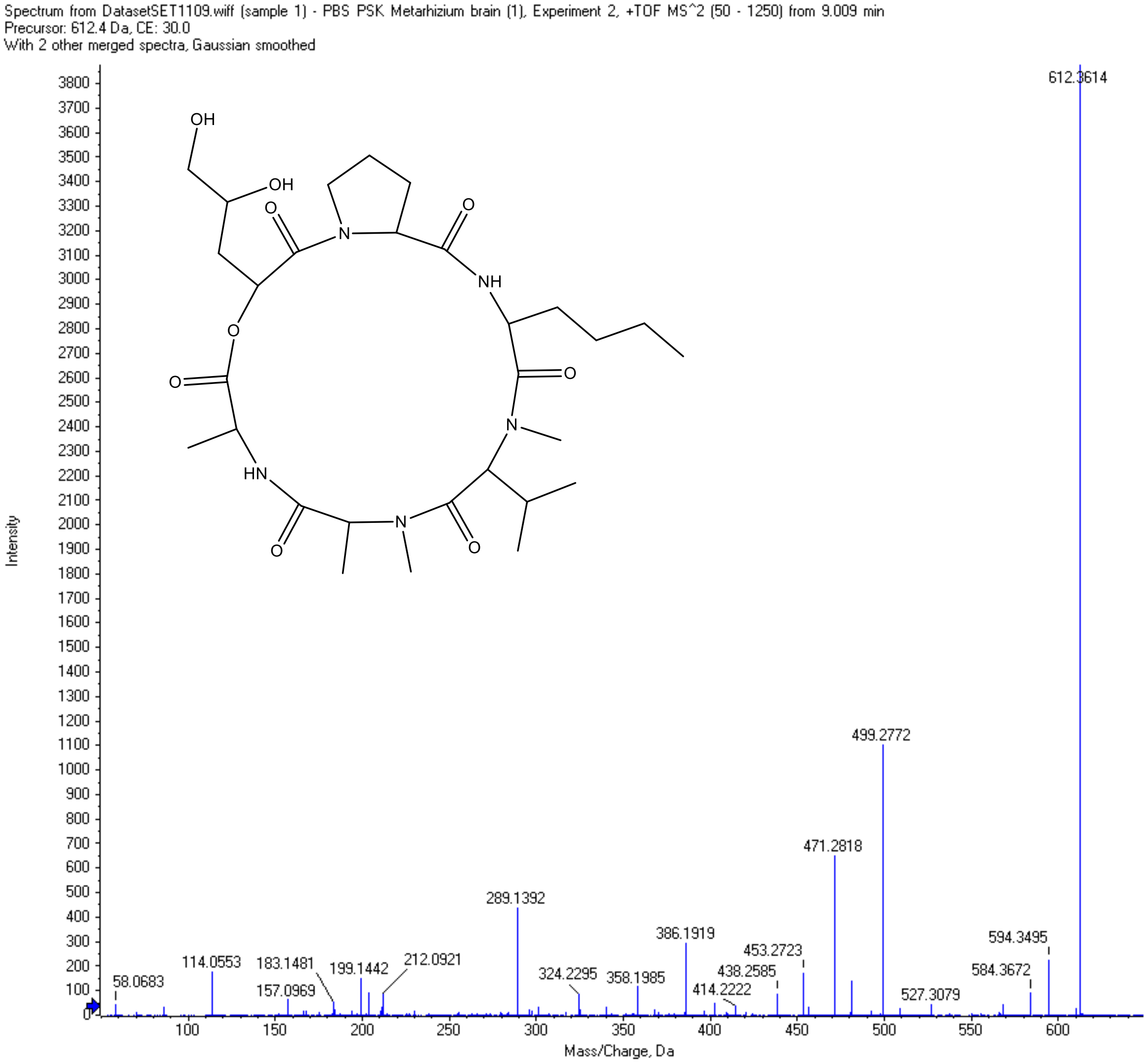

Spectrum from DatasetSET1109.wiff (sample 1) - PBS PSK Metarhizium brain (1), Experiment 2, +TOF MS^2 (50 - 1250) from 9.009 min
Precursor: 612.4 Da, CE: 30.0
With 2 other merged spectra, Gaussian smoothed
Intensity
Mass/Charge, Da
612.3614
499.2772
471.2818
289.1392
386.1919
453.2723
438.2585
414.2222
358.1985
324.2295
594.3495
584.3672
527.3079
114.0553
183.1481
199.1442
212.0921
157.0969
58.0683
OH
OH
O
O
N
NH
O
O
N
HN
O
N
O
O

Spectrum from DatasetSET1109.wiff (sample 1) - PBS PSK Metarhizium brain (1), Experiment 2, +TOF MS^2 (50 - 1250) from 9.969 min
Precursor: 624.4 Da, CE: 30.0
With 2 other merged spectra, Gaussian smoothed

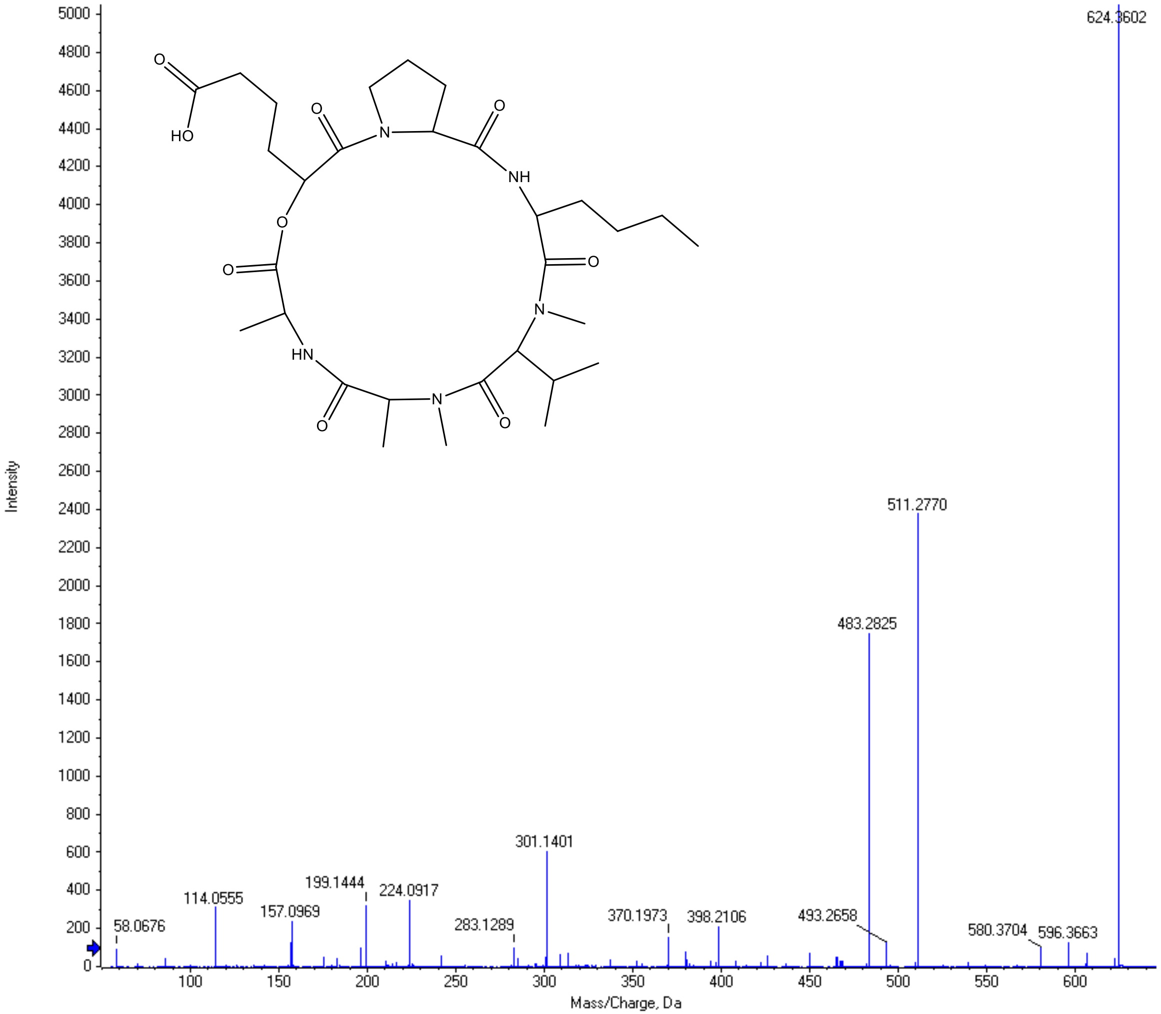

Supplement: Data File S4 — (PDF) [file pone.0070609.s006.pdf]
